# Catalytic Synthesis of (S)-CHBE by Directional Coupling and Immobilization of Carbonyl Reductase and Glucose Dehydrogenase

**DOI:** 10.3390/biom14040504

**Published:** 2024-04-21

**Authors:** Yadong Wang, Ruiqi Sun, Peng Chen, Fenghuan Wang

**Affiliations:** 1 Key Laboratory of Geriatric Nutrition and Health, Ministry of Education, Beijing Technology and Business University (BTBU), Beijing 100048, China; wangyadong@btbu.edu.cn (Y.W.); srqsp2017@163.com (R.S.); cp15212120947@163.com (P.C.); 2 School of Light Industry Science and Engineering, Beijing Technology and Business University (BTBU), Beijing 100048, China

**Keywords:** tag/catcher system, coenzyme regeneration, multi-enzyme cascade reaction, enzyme immobilization

## Abstract

Ethyl (S)-4-chloro-3-hydroxybutyrate ((S)-CHBE) is an important chiral intermediate in the synthesis of the cholesterol-lowering drug atorvastatin. Studying the use of SpyTag/SpyCatcher and SnoopTag/SnoopCatcher systems for the asymmetric reduction reaction and directed coupling coenzyme regeneration is practical for efficiently synthesizing (S)-CHBE. In this study, Spy and Snoop systems were used to construct a double-enzyme directed fixation system of carbonyl reductase (BsCR) and glucose dehydrogenase (BsGDH) for converting 4-chloroacetoacetate (COBE) to (S)-CHBE and achieving coenzyme regeneration. We discussed the enzymatic properties of the immobilized enzyme and the optimal catalytic conditions and reusability of the double-enzyme immobilization system. Compared to the free enzyme, the immobilized enzyme showed an improved optimal pH and temperature, maintaining higher relative activity across a wider range. The double-enzyme immobilization system was applied to catalyze the asymmetric reduction reaction of COBE, and the yield of (S)-CHBE reached 60.1% at 30 °C and pH 8.0. In addition, the double-enzyme immobilization system possessed better operational stability than the free enzyme, and maintained about 50% of the initial yield after six cycles. In summary, we show a simple and effective strategy for self-assembling SpyCatcher/SnoopCatcher and SpyTag/SnoopTag fusion proteins, which inspires building more cascade systems at the interface. It provides a new method for facilitating the rapid construction of in vitro immobilized multi-enzyme complexes from crude cell lysate.

## 1. Introduction

(S)-Ethyl-4-chloro-3-hydroxybutanoate ((S)-CHBE), a key chiral intermediate widely used in synthesizing various active pharmaceuticals, plays a crucial role in preparing the side chain of the cholesterol-lowering drug atorvastatin (Lipitor) [1]. In addition, (S)-CHBE is crucial for synthesizing Slagenins B and C and 1,4-dihydropyridine-type β-blockers [2], prompting significant research efforts toward preparing optically active CHBE.

(S)-CHBE can be efficiently prepared by racemate splitting and asymmetric catalysis [3]. Chiral splitting using a biological method to obtain optically pure CHBE from the racemic ethyl 4-chloro-3-hydroxybutyrate has become a promising approach. While the biological splitting method offers mild conditions, excellent stereoselectivity, and simplified isolation, its 50% theoretical yield and low atom utilization rate limit its efficiency [4]. In contrast, asymmetric catalytic reduction offers more significant economic and efficiency benefits [5]. This approach directly converts a latent chiral substrate, like ethyl 4-chloroacetoacetate (COBE), into a single-configuration chiral product (CHBE) through the asymmetric reduction reaction mediated by oxidoreductases, avoiding the need for a separate splitting step [6,7]. This method not only has a high atom utilization rate but also increases the substrate concentration dramatically and achieves a 100% theoretical yield. Numerous studies have explored asymmetric reduction for CHBE production, primarily utilizing short-chain dehydrogenases as biocatalysts. For example, Tan et al. [8] used short-chain dehydrogenase SgCR for asymmetric reduction of COBE under optimized reaction conditions and obtained a space-time yield of (S)-CHBE of 22.5 g^−1^L^−1^h^−1^ and a 99% e.e. value (e.e. serves as a metric for the optical purity of a chiral compound). Wang et al. [9] isolated an (S)-selective short-chain dehydrogenase, CPE, from *Candida parapsilosis* CDC317. They coupled this enzyme with GDH to catalyze the conversion of COBE to (S)-CHBE, achieving a yield of 91% and an e.e. value of 99%. Recently, more and more studies have also found that aldo-keto reductases can be used for the asymmetric synthesis of CHBE. For example, Wang et al. [10] cloned the aldo-keto reductase LEK from a strain of *L. elongisporus* NRRL YB-4239 and molecularly modified it. They selected the mutant enzyme LEKS209G co-expressed with GDH in *E. coli* to catalyze the generation of (R)-CHBE from COBE with a spatiotemporal yield of 56.51 mmol L^−1^h^−1^ and an e.e. value of the product up to 99%. These studies not only provide a new route for the asymmetric synthesis of CHBE but also provide more options for the preparation of chiral drugs.

Despite the many advantages of the asymmetric reduction method, its disadvantage lies in the fact that oxidoreductases rely on the coenzyme NAD(P)H as a hydrogen and electron transfer medium when performing their functions [11,12], requiring the addition of exogenous coenzymes. However, nicotinamide coenzymes are expensive, unstable, and difficult to reuse, resulting in high costs and limiting their industrial applications [13,14]. In order to optimize the efficiency of coenzymes and improve the biosynthesis of chiral alcohols, researchers introduced advanced techniques such as multi-enzyme systems, substrate coupling, and enzyme coupling [15,16,17]. Therefore, we planned to utilize glucose dehydrogenase (GDH) to regenerate NADPH to provide hydrogen atoms for the carbonyl reductase-mediated COBE asymmetric reduction reaction. The instability and difficulty of reusing free enzymes are significant challenges during coenzyme regeneration. Establishing a stable and efficient immobilized enzyme-coupled catalytic system was crucial to address these challenges. Several studies reported coenzyme regeneration by co-immobilizing double (or multiple) enzymes [18,19]. For instance, Peng et al. [20] co-immobilized double enzymes (2,3-butanediol dehydrogenase and formate dehydrogenase) on functionalized silica nanoparticles, which not only regenerated coenzymes but also realized the regeneration of (R)-1-phenyl-1,2-ethylene glycol chiral production. Multi-enzyme immobilization technology, as an extension of single-enzyme immobilization, further enhances the stability of catalysts, especially in the face of temperature, organic solvent, and pH changes. As this technology advances, it shows promising applications in various fields, such as biosensors, eliminating equilibrium inhibition, and coenzyme regeneration. Several strategies have been developed for multi-enzyme immobilization, including random co-immobilization, sequential co-immobilization, localized immobilization, and point-specific co-immobilization [21]. These strategies enrich the means of multi-enzyme immobilization and provide more possibilities for their application in different fields.

Recently, multi-enzyme cascades have attracted great interest in the field of biotransformation. This reaction can efficiently complete the complex biotransformation process through the synergistic action of multiple enzymes. In order to construct an efficient and stable multi-enzyme cascade reaction system, researchers have proposed various strategies [22]. Protein scaffold assembly was considered a method with great potential for industrial applications due to its programmability, controllability, simplicity and cost-effectiveness. The SpyCatcher/SpyTag system, based on the CnaB2 structural domain of FbaB, formed stable isopeptide bonds under various conditions. Asp in SpyTag and Lys in SpyCatcher can spontaneously generate covalent bonds under various temperatures, pH levels and buffers. Therefore, the SpyTag/SpyCatcher system has essential potential as a multi-enzyme self-assembly tool [23,24]. In addition, similar systems, SnoopCatcher and SnoopTag, were developed based on the D4 structural domain of the *Streptococcus pneumoniae* adhesion trichome protein RrgA [25,26]. Spy and Snoop systems offered high specificity and stability. They enabled orthogonal reactions, which allowed the simultaneous use of multiple assembly strategies within a single system, producing highly modular multiprotein complexes with vast potential for various biotechnological applications [27]. Over the past decades, the system has been used in various fields, such as enzyme immobilization, protein labeling, protein hydrogel preparation, and immunotherapy, while also showing great potential in multi-enzyme assembly. For example, Bao [28] and colleagues successfully assembled three cascade enzymes onto bacterial biofilm using SpyTag/SpyCatcher and SnoopTag/SnoopCatcher to achieve enzyme programming on the biofilm surface. Despite the unique advantages shown by this strategy, potential issues of expression, membrane translocation, and assembly of the fusion proteins need to be further explored to facilitate their extensive application in practice. Lin [29] and his team have proposed a green multi-enzyme co-immobilization strategy that combines elastin-like polypeptide (ELP)-mediated biomimetic silicification and SpyCatcher/SpyTag spontaneous covalent bond formation. The SpyTag-labeled recombinant duplex xylanase-ST-lichenase (XSTL) was self-coupled to the nanocarriers (SCE@SiO2) without additional surface modifications and cross-linkers. The method may have problems with protein misfolding and the formation of inclusion bodies, adversely affecting the final enzyme activity and stability. Therefore, some studies considered using orthogonal reactive protein pairs to fuse the different enzymes with the vector. Notably, Wei [30] assembled three terpene biosynthetic enzymes in *E. coli* using tobacco mosaic virus (TMV) virus-like particles (VLPs) as protein scaffolds in combination with orthogonal reaction protein pairs (SpyCatcher/SpyTag and SnoopCatcher/SnoopTag) as linker modules. This not only demonstrates the synergistic biosynthetic ability of the SpyCatcher-SpyTag/SnoopCatcher-SnoopTag orthogonal system in a multi-enzyme complex system but also proves its great potential to improve intracellular metabolic fluxes and increase product yields.

In this study, carbonyl reductase (BsCR) and glucose dehydrogenase (BsGDH) from *Bacillus subtilis* (strain 168) were fusion-expressed with two tagged proteins, SpyTag and SnoopTag, respectively, by using genetic engineering techniques. Through the autocatalytic binding of Tag and Catcher proteins, BsCR and BsGDH were co-immobilized on epoxy resin to form immobilized enzymes. The immobilized enzymes were characterized using field emission scanning electron microscopy (SEM) and Fourier transform infrared spectroscopy (FTIR). Subsequently, the enzymatic properties of the immobilized enzyme were investigated, the reaction conditions for the double-enzyme immobilization system were optimized, and the operational stability was tested. This study achieved the directional coupling and immobilization of redox reaction and coenzyme regeneration, laying the foundation for the industrial production and application of (S)-CHBE.

## 2. Materials and Methods

### 2.1. Materials

Plasmid pET28a, *Bacillus subtilis* 168, host cell *E. coli* DH5α, *E. coli* BL21 (DE3), and Rosetta (DE3) were kept in the laboratory. Restriction endonuclease, T4 DNA ligase, and PrimeSTAR HS DNA polymerase for fragment amplification were purchased from Takara Biomedical Technology (Beijing, China). The plasmid extraction kit and gel recovery kit were purchased from OMEGA Biotek. Ni column His Trap HP (17-5248-01) was purchased from GE Healthcare. A 0.22 μm filter membrane was purchased from Merck Millipore (USA). Seamless cloning and BCA protein assay kit were purchased from Beyotime Biotechnology (Shanghai, China). Nicotinamide adenine dinucleotide phosphate (NADP) and NADPH were purchased from Baoruyi (Beijing, China). Epoxy resin LXTE-600 was provided by Lanxiao Technology Co. All other reagents were domestic analytical purities. All primers used in this study were synthesized by Huada Gene Technology Co., (Beijing, China).

LB medium was used to culture recombinant *E. coli*, constituted as follows (mass fraction): tryptone 1%, yeast extract 0.5%, sodium chloride 1%, agar powder 1.5% (solid medium); pH was adjusted to 7.0. For use, kanamycin was added to a final concentration of 50 μg/mL and chloramphenicol to a final concentration of 5 μg/mL.

### 2.2. Construction of Plasmids

The carbonyl reductase gene *yueD* (GenBank accession no. 936558) and glucose dehydrogenase gene *bsgdh* (GenBank accession no. MG425967.1) were obtained from *Bacillus subtilis* (strain 168). The *yueD* and *bsgdh* genes were amplified by polymerase chain reaction (PCR) from the Bacillus subtilis strain 168 genome using specific primers: yueD-F and yueD-R for *yueD* and bs-F and bs-R for *bsgdh*. Following PCR, the amplified products *yueD* and *bsgdh* were purified using 1% (*w*/*v*) agarose gel electrophoresis. The plasmid pET28a and the purified *yueD* and *bsgdh* were then individually digested with the restriction enzymes BamHI and XhoI at 37 °C for 3 h. The digestion success was verified by agarose gel electrophoresis, and the desired fragments were gel-purified. Subsequently, the purified *yueD* and *bsgdh* fragments were ligated independently into the pET-28a vector using T4 DNA ligase at 16 °C overnight. Finally, the ligation reactions were transformed into *E. coli* DH5α receptor cells to obtain the recombinant plasmids pET28a-*yueD* and pET28a-*bsgdh*.

The plasmids pET28a-VLT and pET28a-DLSN, previously constructed in our laboratory, included *Linker-SpyTag* and *Linker-SnoopTag* gene fragments, respectively (Figure S1a,b). We obtained the *Linker-SpyTag* gene fragment by PCR amplification using primers YSTY56-F and YSTY44-R with the pET28a-VLT plasmid as a template. Similarly, the *Linker-SnoopTag* gene fragment was amplified using primers GSTY44-F and GSTY44-R with pET28a-DLSN as a template. The sequence information for *Linker-SpyTag* and *Linker-SnoopTag* is shown in Tables S1 and S2. Subsequently, plasmids pET28a-*yueD* and pET28a-*bsgdh* were digested with restriction enzyme XhoI at 37 °C for 3 h, and purified by gel recovery. The digested plasmid pET28a-*yueD* was ligated with the PCR product *Linker-SpyTag* by homologous recombination using the Seamless Cloning Kit to obtain the recombinant plasmid pET28a-*ydlspt*. Similarly, the enzymatically cleaved plasmid pET28a-*bsgdh* was ligated to the PCR product *Linker-SnoopTag* by homologous recombination to obtain the recombinant plasmid pET28a-*bsgdhsnpt*. The recombinant plasmids were transformed into the cloning host *E. coli* DH5 α receptor cells by heat shock at 42 °C. Transformants were selected on LB agar plates containing the appropriate antibiotic. Positive clones were identified by colony PCR and subsequently cultured for plasmid extraction. All recombinant plasmids were verified by DNA sequencing and transformed into *E. coli* Rosetta (DE3) for protein expression. This study employed previously constructed recombinant plasmids harboring *SpyCatcher* and *SnoopCatcher* sequences with an N-terminal rigid *PT-Linker*. These plasmids, designated pET28a-His-*PT-SpyCatcher* and pET28a-His-*PT-SnoopCatcher*, were obtained by integrating the respective fragments into the pET28a vector (Figure S1c,d). Detailed information regarding gene sequences for *SpyCatcher* and *SnoopCatcher* with *PT-linker* can be found in Tables S1 and S2, respectively. The sequences of all PCR primers used in this experiment are listed in Table 1. Plasmids are summarized in Table 2.

### 2.3. Protein Expression and Purification

*E. coli* Rosetta (DE3) containing recombinant plasmids were cultured overnight in 3 mL of LB medium (with corresponding resistance) at 37 °C, 220 rpm. The overnight seed solution was transferred 1:100 into LB medium (with corresponding resistance) and incubated at 37 °C and 220 rpm until the OD600 was 0.6–0.8. Protein expression was induced by adding IPTG to a final concentration of 0.5 mM, followed by incubation at 16 °C for 16–20 h. Cells were harvested by centrifugation (8000 rpm, 4 °C, 10 min), washed with cold PBS (50 mM, pH 7.4), and resuspended in PBS. Cell lysis was achieved using sonication in an ice-water bath. The bacterial lysates were centrifuged at 8000 rpm for 20 min at 4 °C, and the supernatant was collected and filtered through an aqueous-based microporous membrane (0.22 μm) to obtain a crude protein solution. The recombinant protein containing His×6-Tag was purified by nickel affinity chromatography, and the Ni-NTA column was pre-equilibrated by binding buffer (20 mM Na_2_HPO_4_, 20 mM NaH_2_PO_4_, 500 mM NaCl, 20 mM imidazole, pH 7.4). The crude protein solution was added to the Ni column, and the unbound proteins were washed away. The recombinant protein was eluted using elution buffer (20 mM Na_2_HPO_4_, 20 mM NaH_2_PO_4_, 500 mM NaCl, 500 mM imidazole, pH 7.4). The eluted protein was desalted using a dextran gel column (HiTrapTM desalting, 5 mL) equilibrated with desalting buffer (2.7 mM KCl, 4.4 mM KH_2_PO_4_, 10 mM Na_2_HPO_4_, 137 mM NaCl). The protein was loaded onto the column and eluted with desalting buffer at a flow rate of 2 mL/min. Purified desalted recombinant proteins were analyzed using sodium dodecyl sulfate-polyacrylamide gel electrophoresis (SDS-PAGE). Protein concentration was determined by the BCA method on an enzyme marker.

### 2.4. Connection Validation and Enzyme Immobilization

In vitro enzymatic ligation was performed to assess Catcher-Tag complex formation. Equal volumes (1:1) of purified SpyCatcher and BsCR-SpyTag crude extract were mixed and incubated at 37 °C for 30 min. Similarly, purified SnoopCatcher was combined with BsGDH-SnoopTag crude extract in a 1:1 (*v*/*v*) ratio and incubated under the same conditions. Then, SDS-PAGE was used to confirm tag ligation to the respective catcher. 

Epoxy resin LXTE-600 was weighed and washed three times with 0.1 M PBS buffer (pH 7.4). Purified SpyCatcher or SnoopCatcher was then added to the LXTE-600 and gently mixed for 12 h at 25 °C on a shaker. The solid carrier (LXTE@SpyCatcher or LXTE@SnoopCatcher) was subsequently separated by centrifugation. To block any remaining reactive epoxy groups on the carrier, the resin was incubated with 3 M glycine solution (pH 8.5) for 24 h at room temperature. Finally, the LXTE@SpyCatcher/SnoopCatcher was isolated by centrifugation and washed three times with 0.1 M PBS buffer (pH 7.4) to remove unbound protein and excess glycine solution.

For enzyme immobilization, either LXTE@SpyCatcher or LXTE@SnoopCatcher resin was incubated with the corresponding crude enzyme solution (BsCR-SpyTag for SpyCatcher and BsGDH-SnoopTag for SnoopCatcher) in a shaker at 16 °C for 4 h. Following incubation, the mixture was centrifuged, and the supernatant was carefully removed using a pipette to isolate the immobilized enzyme fraction. The immobilized enzyme was then washed five times with 0.1 M PBS buffer (pH 7.4) to remove unbound materials. This process yielded the immobilized enzymes, LXTE@BsCR-SpyTag and LXTE@BsGDH-SnoopTag, which were then ready for enzyme activity assays.

Protein immobilization efficiency was assessed by analyzing the amount of protein in the initial solution and the amount of protein in the solution after immobilization. The amount of protein in solution was quantified by SDS-PAGE of the proteins and grayscale analysis of the target protein bands using ImageJ software. The immobilization efficiency was calculated as follows:

Protein immobilization (mg) = Total supernatant protein before immobilization (mg) − Total supernatant protein after immobilization (mg)(1)

(2) $$Protein\;immobilization\;efficiency\;(\%)=\frac{Protein\;immobilization\;amount\;(mg)}{Total\;supernatant\;protein\;amount\;before\;immobilization\;(mg)}$$

Based on the above immobilization method, the process conditions for immobilizing purified SpyCatcher/SnoopCatcher on the LXTE-600 vector were investigated. Optimized parameters include protein uploading volume (mg/(g epoxy resin)) and fixation time (0–12 h).

### 2.5. SEM Analysis

The samples were freeze-dried, fixed on a conductive adhesive, and sprayed with a nanogold coating. The morphology of the resins was observed by field emission scanning electron microscopy (SEM) (Zeiss Sigma300, Oberkochen, Germany). SEM images were taken using an in-lens detector operating at an accelerating voltage of 2 kV.

### 2.6. Secondary Structure Analysis

The epoxy and enzyme immobilized resins were confirmed using Fourier transform infrared spectroscopy (FTIR) (ALPHA II, Bruker, Germany).

### 2.7. Determination of Enzyme Activity

The enzyme activities of BsCR, BsCR-SpyTag, and LXTE@BsCR-SpyTag were determined by measuring the decrease in absorbance value at 340 nm during the reaction by UV spectrophotometer. The enzyme activity assay system was 100 mM PBS buffer (pH 7.4), 5 mM COBE, 0.2 mM NADPH, and the appropriate amount of enzyme. The PBS buffer, substrate, and enzyme were added sequentially, mixed well, and then water-bathed at a certain reaction temperature for 3 min. NADPH was added quickly and the decrease in absorbance at 340 nm was measured, while the control group used buffer solution instead of enzyme solution. The enzyme activity unit was defined as the amount of enzyme required to consume 1 μmol of NADPH per minute under optimal reaction conditions as one enzyme activity unit (U), i.e., 1 U = 1 μmol/min.

The enzyme activities of BsGDH, BsGDH-SnoopTag, and LXTE@BsGDH-SnoopTag were determined by measuring the increase in absorbance at 340 nm during the reaction by a UV spectrophotometer. The enzyme activity assay system was Tris-HCl buffer (100 mM, pH 8.0), 0.1 M glucose, 2 mM NADP^+^, and the appropriate amount of enzyme. The buffer, substrate, and enzyme solution were added sequentially, mixed well, and then water-bathed for 3 min at a certain reaction temperature. Then, NADP^+^ was added rapidly, and the increase in absorbance at 340 nm was measured. The control group used a buffer solution instead of an enzyme solution. Definition of enzyme activity unit: Under optimal reaction conditions, the amount of enzyme required to generate 1 μmol of NAD(P) per minute is one enzyme activity unit (U), i.e., 1 U = 1 μmol/min.

All the above experiments were performed in triplicate and the mean values were calculated based on three independent experiments with standard deviation.

### 2.8. Effect of pH and Temperature on the Activity of BsCR and BsGDH

To study the effect of different pH levels on the activity of free and immobilized BsCR (or BsGDH), the enzyme activity was measured at pH values of 5.0, 5.5, 6.0, 6.5, 7.0, 7.5, 8.0, 8.5, and 9.0, at a reaction temperature of 35 °C. Other conditions were kept constant, and the reaction was carried out after changing the buffer of the reaction system from 2.7 to a buffer of different pH. The experiment was repeated three times, and the relative enzyme activity was plotted against ambient pH using the highest enzyme activity detected, which was 100% of the relative enzyme activity. To investigate the effects of different temperatures on the activities of free and immobilized BsCR (or BsGDH), the individual components were added according to the reaction system given in Section 2.7, keeping other conditions unchanged. The activity of the enzymatic reactions was detected at the temperatures of 25 °C, 30 °C, 35 °C, 40 °C, 45 °C, 50 °C, 55 °C, and 60 °C. The experiment was repeated three times, and the relative enzyme activity was plotted against temperature using the highest enzyme activity detected, which was 100% of the relative enzyme activity.

### 2.9. pH and Temperature Stability Analysis of Free Enzymes and the Immobilized Enzymes

BsCR-SpyTag, BsGDH-SnoopTag, LXTE@BsCR-SpyTag and LXTE@BsGDH-SnoopTag were incubated in different pH buffers (5.0, 6.0, 7.0, 8.0, 9.0) at 4 °C for 24 h. Samples were taken at regular intervals, and enzyme activity was detected at a reaction temperature of 35 °C with COBE and glucose as substrates. The enzyme activity was plotted against incubation time using the enzyme activity detected before incubation as 100% of the relative enzyme activity. BsCR-SpyTag, BsGDH-SnoopTag, LXTE@BsCR-SpyTag and LXTE@BsGDH-SnoopTag were incubated at 25 °C, 30 °C, 35 °C, 40 °C and 45 °C for 24 h. Samples were taken at regular intervals, and enzyme activity was measured under standard conditions using COBE and glucose as substrates. The relative enzyme activity was plotted against incubation time using the enzyme activity detected before incubation as 100% of the relative enzyme activity.

### 2.10. Asymmetric Reduction Reactions of COBE

We used PBS buffer (0.1 M) at pH 7.0 as the reaction medium, while NADPH (0.2 mM), NADP^+^ (0.2 mM), glucose (100 mM), and COBE (40 mM) were added as the substrates. The BsCR/BsGDH mixture was added to the reaction system to catalyze the reaction. During the reaction, we controlled the reaction temperature and buffer pH and performed continuous rotation at 70 rpm to ensure the efficient performance of the reaction. After 8 h of reaction, we removed the sample and accurately determined the yield of (S)-CHBE by high-performance liquid chromatography (HPLC).

### 2.11. Effect of Different Conditions on the Yield of (S)-CHBE

A double-enzyme system was applied to convert COBE to (S)-CHBE for coenzyme regeneration. We set several substrate concentration gradients, including 10, 20, 30, 40, 50, and 60 mM, to explore the optimal substrate concentration. We performed the catalytic reaction at 15, 20, 25, 30, 35, 40, and 45 °C for the reaction temperature to find the optimal reaction temperature. Similarly, different reaction pH values (pH 5.0, 6.0, 7.0, 8.0, and 9.0) were set to observe the effect on the catalytic effect. Each reaction was carried out according to the system described in Section 2.10, and the supernatant was taken after the reaction to accurately determine the yield of (S)-CHBE by HPLC. The experiments were repeated three times.

### 2.12. Reusability of the Immobilized Enzyme

The reusability of the immobilized enzyme was studied for eight cycles. The reaction time for each cycle was 1 h, and the reaction was carried out under optimal conditions. The supernatant of each cycle was collected for HPLC analysis. The experiment was repeated three times. The immobilized enzyme was washed using PBS buffer (50 mM, pH 7.5) and proceeded to the next catalytic cycle. Each cycle was performed in PBS buffer (50 mM, pH 8.0): 20 mM COBE, 40 mM glucose, 0.2 mM NADP^+^, 30 °C and 70 rpm. The operational stability of the prepared immobilized enzyme was analyzed by determining the amount of (S)-CHBE produced during cycle repetitions in three parallel experiments. The yield of (S)-CHBE produced in the first cycle was 100%.

### 2.13. HPLC Analysis of (S)-CHBE

The yield of (S)-CHBE was analyzed by HPLC using an Agilent LC 1260 Infinity II: the column was an Agilent TC-C18 (packing size of 5 μm, column length of 250 mm × column inner diameter of 4.6 mm) with an injection volume of 10 μL, and the finalized mobile phases were selected as 80% water (0.1% formic acid) and 20% acetonitrile. The column temperature was set at 25 °C, the flow rate was 0.8 mL/min, and the wavelength of the VWD detector was 220 nm. The retention time of (S)-CHBE was 15.9 min.

The extent of the reaction was expressed by the yield (chemical yield, %), defined as follows:

(3) $$Yield=\frac{Final\;product\;concentration}{Initial\;substrate\;concentration}\times 100\%$$

## 3. Results

### 3.1. Recombinant Protein Expression and Purification

**Figure 1:**
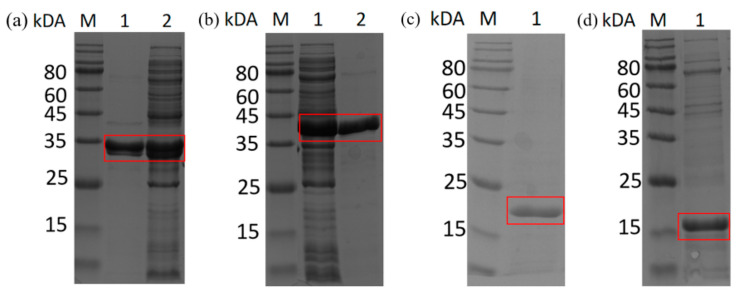
SDS-PAGE analysis of recombinant fusion protein expression in *E. coli*. (**a**) M: Protein Marker; lane 1: purified BsCR-SpyTag enzyme solution; lane 2: BsCR-SpyTag crude enzyme solution. (**b**) M: Protein Marker; lane 1: BsGDH-SnoopTag crude enzyme solution; lane 2: purified BsGDH-SnoopTag enzyme solution. (**c**) M: Protein Marker; lane 1: purified SpyCatcher protein solution. (**d**) M: Protein Marker; lane 1: purified SnoopCatcher protein solution. The bands corresponding to BsCR-SpyTag, BsGDH-SnoopTag, SpyCatcher, and SnoopCatcher are marked with red boxes.

The constructed recombinant vectors pET28a-*SpyCatcher*, pET28a-*SnoopCatcher*, pET28a-*yueD*, pET28a-*bsgdh*, pET28a-*yueD-SpyTag*, and pET28a-*bsgdh-SnoopTag* were induced to be expressed in *E. coli*. The collected recombinant cells were broken and centrifuged. Then, the supernatant was purified using a Ni-NTA column, and the purified protein solution was desalted. Expression of the recombinant proteins was confirmed using SDS-PAGE (Figure 1 and Figure S2). As expected, six recombinant proteins were obtained: SpyCatcher (16.1 kDa), SnoopCatcher (15.36 kDa), BsCR (30.65 kDa), BsCR-SpyTag (33.75 kDa), BsGDH (31.63 kDa) and BsGDH-SnoopTag (34.37 kDa).

### 3.2. Optimization of the Enzyme Immobilization Process

#### 3.2.1. Catcher and Tag Connection Validation

**Figure 2:**
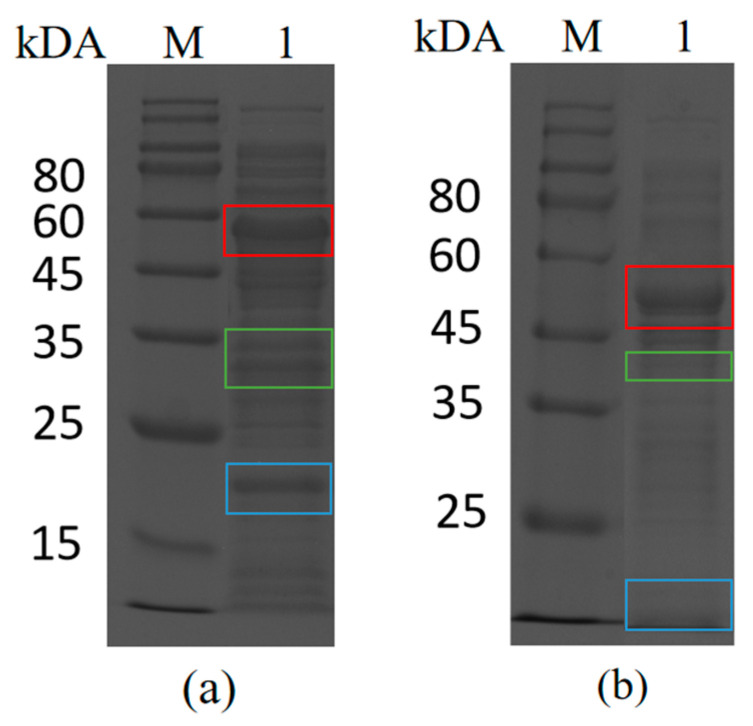
SDS-PAGE analysis of Catcher and Tag self-assembly in vitro. (**a**) M: Protein Marker; lane 1: mixture after ligation reaction of purified SpyCatcher with BsCR-SpyTag crude enzyme solution. The bands corresponding to SpyCatcher, BsCR-SpyTag, and linker proteins are marked with colored boxes (SpyCatcher: blue box; BsCR-SpyTag: green box; Linker protein: red box). (**b**) M: Protein Marker; lane 1: mixture after ligation reaction of purified SnoopCatcher with BsGDH-SnoopTag crude enzyme solution. The bands corresponding to SnoopCatcher, BsGDH-SnoopTag, and linker proteins are marked with colored boxes (SnoopCatcher: blue box; BsGDH-SnoopTag: green box; Linker protein: red box).

The following experiments were carried out to confirm that isopeptide bonds could be formed between Catcher and Tag by spontaneous reaction for the directed immobilization process. The purified SpyCatcher was mixed with crude enzyme BsCR-SpyTag for the reaction, and the reaction products were detected using SDS-PAGE. As shown in Figure 2a, apart from the original protein band of the crude enzyme BsCR-SpyTag and the purified SpyCatcher band, a new protein band (52.23 kDa) appeared in the reaction product. The molecular weight of the new band corresponds precisely to the theoretically predicted size of the combined protein (SpyCatcher-BsCR-SpyTag). Similar results were observed in the Snoop system (Figure 2b). The results showed that spontaneous binding occurs between SpyCatcher and BsCR-SpyTag as well as SnoopCatcher and BsGDH-SnoopTag. Therefore, the Spy and Snoop systems could play a vital role in the targeted immobilization of enzymes as efficient and reliable protein-protein linking tools.

#### 3.2.2. Enzyme-Directed Immobilization on Epoxy Resin

**Figure 3:**
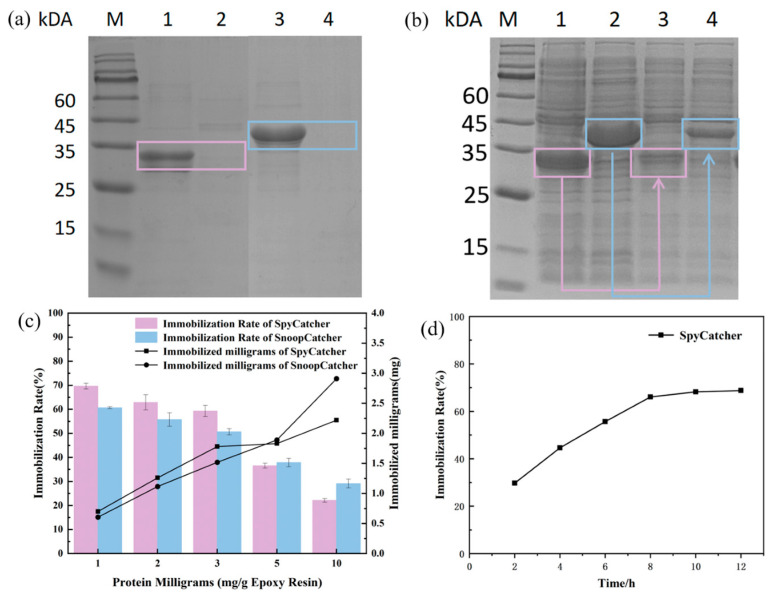
Optimization of immobilization conditions. (**a**) SDS-PAGE results of immobilization of purified SpyTag and SnoopTag fusion proteins; M: Protein Marker; lane 1: Purified BsCR-SpyTag; lane 2: Protein supernatant after targeted immobilization of purified BsCR-SpyTag with LXTE@SpyCatcher; lane 3: Purified BsGDH-SnoopTag; lane 4: Protein supernatant after targeted immobilization of purified BsGDH-SnoopTag with LXTE@SnoopCatcher. The bands corresponding to BsCR-SpyTag and BsGDH-SnoopTag are marked with colored boxes (BsCR-SpyTag: pink box; BsGDH-SnoopTag: blue box). (**b**) SDS-PAGE results of SpyTag and SnoopTag fusion proteins before and after immobilization; M: Protein Marker; lane 1: BsCR-SpyTag crude enzyme solution; lane 2: BsGDH-SnoopTag crude enzyme solution; lane 3: Protein supernatant after targeted fixation of BsCR-SpyTag crude enzyme solution with LXTE@SpyCatcher; lane 4: Protein supernatant after targeted fixation of BsGDH-SnoopTag crude enzyme solution with LXTE@SnoopCatcher protein supernatants after fixation. The bands corresponding to BsCR-SpyTag and BsGDH-SnoopTag are marked with colored boxes (BsCR-SpyTag: pink box; BsGDH-SnoopTag: blue box). (**c**) Relationship between different inputs of SpyCatcher and SnoopCatcher and immobilization efficiency and immobilization amount; (**d**) Relationship between SpyCatcher immobilization time and immobilization efficiency.

Epoxy resin was selected as the carrier for enzyme immobilization due to its cost-effectiveness and wide application in industrial applications. To investigate the amount and efficiency of catcher immobilization, we conducted separate experiments. Different quantities of purified SpyCatcher or SnoopCatcher were added per gram of epoxy resin. The catcher proteins were then immobilized on the epoxy resin through covalent bonding. The immobilized products were called LXTE@SpyCatcher and LXTE@SnoopCatcher. Following immobilization, the supernatant was analyzed by SDS-PAGE, revealing bands corresponding to the unimmobilized Catcher protein (Figure S3). We measured the protein concentration before and after fixation by BCA to extrapolate the amount of protein in the immobilized catcher and the efficiency of immobilization. The results showed that the immobilization efficiency of SpyCatcher was nearly 70% when 1 mg of protein was added. However, increasing the catcher protein input resulted in a rise in immobilized protein quantity but decreased immobilization efficiency (Figure 3c). The immobilization pattern of SnoopCatcher was consistent with that of the SpyCatcher. The SpyCatcher and SnoopCatcher amount was set at 3 mg per gram of epoxy resin to maintain high immobilization efficiency, avoid steric hindrance and ensure optimal reactivity. The effect of immobilization time was also investigated. As time increased, the efficiency of immobilization steadily rose. However, when the time reached 8 h, the immobilization efficiency tended to be stable and no longer increased significantly (Figure 3d). Therefore, 8 h was chosen as the optimal immobilization time. Following the ligation of SpyCatcher or SnoopCatcher, the respective epoxy carriers, LXTE@SpyCatcher and LXTE@SnoopCatcher, were capped with glycine. Subsequently, purified BsCR-SpyTag and BsGDH-SnoopTag were immobilized onto their corresponding carriers at a 1:1 molar ratio. The supernatant of the immobilized system was analyzed by SDS-PAGE, revealing significantly weaker bands corresponding to BsCR-SpyTag and BsGDH-SnoopTag compared to the pre-immobilization state. This indicates the successful immobilization of a majority of the enzymes, and we call the immobilized enzymes LXTE@BsCR-SpyTag and LXTE@BsGDH-SnoopTag.

To achieve faster and more convenient enzyme immobilization, we explored the possibility of selectively immobilizing the target enzyme directly from the crude enzyme solution using the LXTE@Catcher carrier. We added the prepared LXTE@SpyCatcher to BsCR-SpyTag crude enzyme solution and LXTE@SnoopCatcher to BsGDH-SnoopTag crude enzyme solution, and gently incubated in a 16 °C shaker for 4 h. Only the BsCR-SpyTag bands showed a significant decrease in intensity in the post-immobilization crude solution, whereas the intensity of other protein bands remained unchanged (Figure 3b). Grayscale analysis (Table S3) revealed an immobilization efficiency of 69.24% for BsCR-SpyTag. The Snoop system showed the same results as the Spy system, with an immobilization efficiency of 69.96% for BsGDH-SnoopTag. These results demonstrated the effectiveness and selectivity of LXTE@Catcher in immobilizing tagged enzymes directly from crude solutions. The particular affinity interaction between SpyCatcher and SpyTag, mediated by a precise molecular recognition mechanism, minimizes interference with or influences the behavior and content of other non-specific proteins in the solution. This selective binding mechanism facilitates efficient enzyme immobilization while minimizing the impact of non-specific proteins on the reaction [31,32]. Consequently, the Catcher and Tag directed coupling strategy, as evidenced by the selective immobilization of BsCR-SpyTag and BsGDH-SnoopTag from the crude enzyme solution, eliminates the purification step in the enzyme immobilization process. This approach facilitates rapid and convenient enzyme immobilization, ultimately saving time and reducing production costs.

After immobilization, we evaluated the enzymatic activity of LXTE@BsCR-SpyTag and LXTE@BsGDH-SnoopTag (Table 3). Both immobilized enzymes exhibited lower activity compared to their free enzymes. LXTE@BsCR-SpyTag showed an activity of 0.669 U/mg, approximately one-third of the free BsCR enzyme activity. Similarly, LXTE@BsGDH-SnoopTag displayed an activity of 2.836 U/mg, representing only one-third of the free BsGDH activity. These observations suggested potential limitations associated with the immobilization process, such as restricted diffusion of substrates to the active sites or conformational changes that might hinder enzyme function. Further optimization of the immobilization protocol may be necessary to improve the activity of immobilized enzymes.

### 3.3. Characterization of Immobilized Enzymes

**Figure 4:**
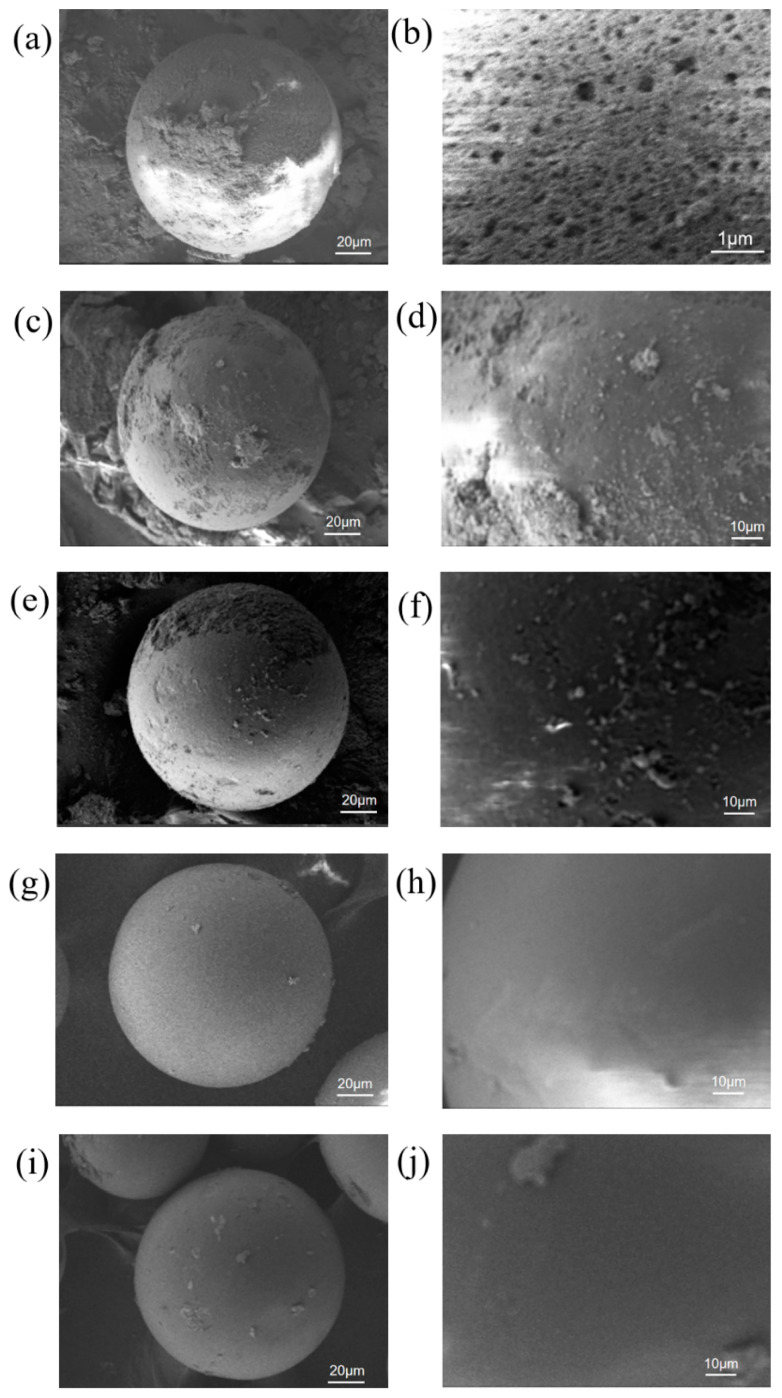
SEM images of the epoxy resin and the immobilized resin. (**a**,**b**) LXTE-600; (**c**,**d**) LXTE@SpyCatcher; (**e**,**f**) LXTE@SnoopCatcher; (**g**,**h**) LXTE@BsCR-SpyTag; (**i**,**j**) LXTE@BsGDH-SnoopTag. (**b**,**d**,**f**,**h**,**j**) were magnified views of the carrier surface.

In order to characterize the immobilized enzymes, we observed the samples after various treatments using scanning electron microscopy (SEM). The results show that LXTE-600, without any modification, exhibited a rough surface with many pores and irregular structures (Figure 4a,b). However, after modification with SpyCatcher and SnoopCatcher, the LXTE@Catcher surfaces became smooth, and the surface holes were significantly reduced (Figure 4c–f). Immobilization of BsCR-SpyTag and BsGDH-SnoopTag resulted in the most dramatic changes in surface morphology (Figure 4g–j). The surfaces of both LXTE@BsCR-SpyTag and LXTE@BsGDH-SnoopTag became much smoother and more continuous, with the pores disappearing completely. These morphological changes visually demonstrated the success of protein immobilization on the carriers and showed that the proteins were evenly and tightly distributed on the carrier surface.

We also performed a Fourier transform infrared spectroscopy (FTIR) analysis of the samples before and after the epoxy resin fixation (Figure S4). The absorption peak intensity at 910 cm^−1^ compared to unmodified epoxy LXTE-600 decreased due to the covalent binding of the epoxy group with the Catcher protein [33]. The characteristic peak variation at 1642 cm^−1^ was due to C=O stretching vibration and N-H stretching and bending vibration in the amide I band, while the absorption peak at 3400 cm^−1^ was related to hydroxyl or amine group vibration [34]. The results indicated that the functional groups of the fixed epoxy carrier were altered, and the enzyme was successfully fixed on the resin.

### 3.4. Characteristics of Free and Immobilized Enzymes

**Figure 5:**
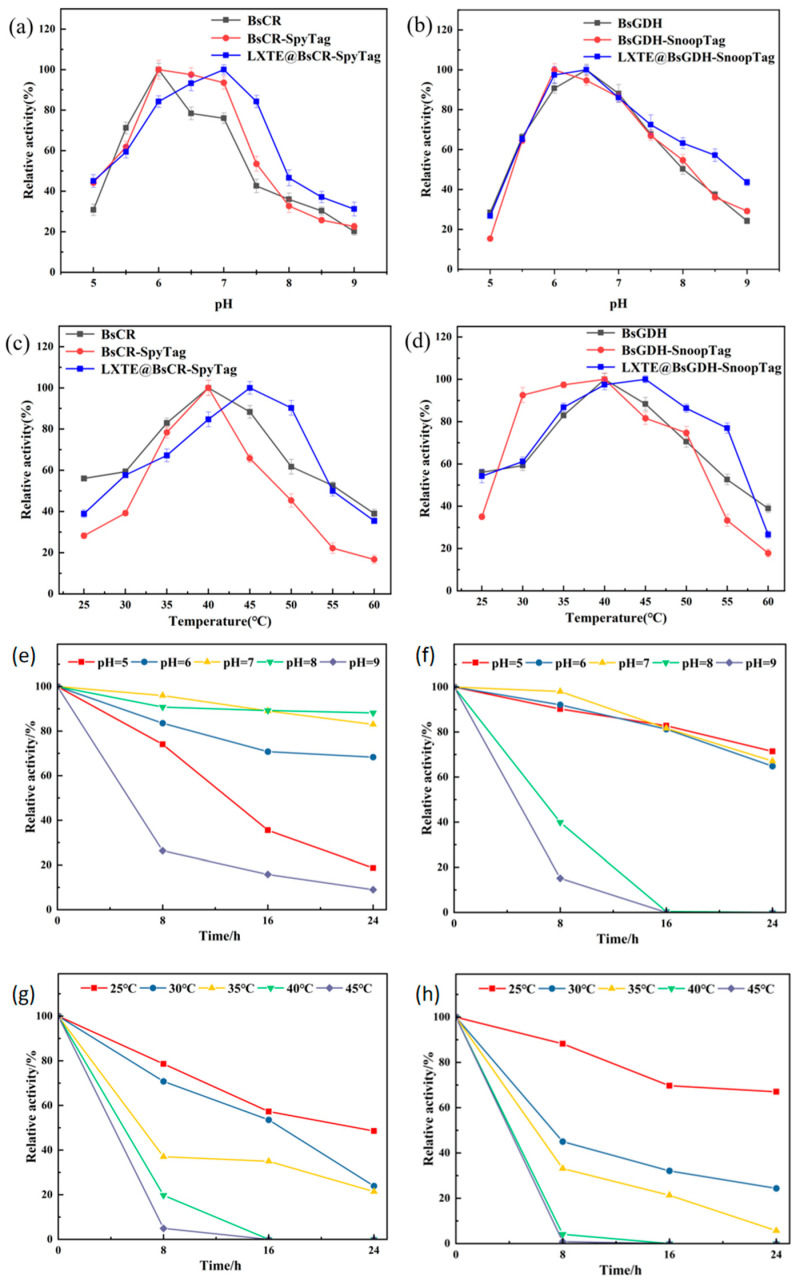
Effect of pH and temperature on catalytic performance of free and immobilized enzymes. (**a**) Effect of pH on the activity of BsCR, BsCR-SpyTag and LXTE@BsCR-SpyTag: pH 5.0–9.0; (**b**) Effect of pH on the activity of BsGDH, BsGDH-SnoopTag and LXTE@BsGDH-SnoopTag: pH 5.0–9.0; (**c**) Effect of temperature on the activity of BsCR, BsCR-SpyTag and LXTE@BsCR-SpyTag: 25–60 °C; (**d**) Effect of temperature on the activity of BsGDH, BsGDH-SnoopTag and LXTE@BsGDH-SnoopTag: 25–60 °C. (**e**) pH stability of LXTE@BsCR-SpyTag: various buffers of pH 5.0–9.0 for 24 h at 4 °C. (**f**) The pH stability of LXTE@BsGDH-SnoopTag: various buffers of pH 5.0–9.0 for 24 h at 4 °C. (**g**) Temperature stability of LXTE@BsCR-SpyTag: 25 °C, 30 °C, 35 °C, 40 °C and 45 °C, pH 7.0, 24 h. (**h**) Temperature stability of LXTE@BsGDH-SnoopTag: 25 °C, 30 °C, 35 °C, 40 °C and 45 °C, pH 7.0, 24 h.

Because temperature and pH significantly affect enzyme activity, we explored the effect of the tag on the optimal temperature and pH of the enzyme. As shown in Figure 5a, both BsCR and BsCR-SpyTag exhibited optimal activity at pH 6.0. Note that at pH 6–7, BsCR-SpyTag showed higher relative activity than BsCR. At the same time, we also observed a difference in pH performance between BsGDH and BsGDH-SnoopTag. As shown in Figure 5b, BsGDH showed the best activity at pH 6.5, while BsGDH-SnoopTag showed the highest activity at pH 6.0. These findings suggest that Tag addition might influence the enzyme’s active center, leading to a shift in the optimal pH. In terms of temperature, the optimal temperature for both BsCR and BsCR-SpyTag was 40 °C (as shown in Figure 5c). When the temperature exceeds this range, the activity of both decreases significantly, especially at 60 °C. This suggests that high temperatures may have damaged the enzyme’s active center or caused changes in its structure. For BsGDH and BsGDH-SnoopTag (Figure 5d), their catalytic activity peaks at 40 °C. Notably, BsGDH-SnoopTag retained high activity between 30–45 °C, suggesting that SnoopTag fusion might enhance enzyme thermal stability.

Building upon the findings with free enzymes, we further explored the properties of the immobilized enzymes. As shown in Figure 5a, the optimal pH for immobilized BsCR was 7, the optimal pH for immobilized BsCR increased by 1 unit compared to free BsCR, and the relative enzyme activity remained above 90% at pH 6.5. These results suggested that immobilization may have altered the enzyme’s active center or its microenvironment, leading to increased pH sensitivity. Similarly, the optimal pH of immobilized BsGDH increased to 7.0, maintaining high enzyme activity in the pH range of 6.0–7.5 (Figure 5b). These results suggested that immobilization can enhance enzyme activity in an alkaline environment. Moreover, the optimum reaction temperature of the immobilized enzymes was also higher than that of the free enzymes. Both immobilized BsCR and immobilized BsGDH had an optimal reaction temperature of 45 °C and maintained high relative activities over a wide temperature range (Figure 5c,d). This suggested that the immobilization process could improve the thermostability of the enzymes or make them more adaptable to changes in temperature. These findings supported the potential advantages of immobilized enzymes in industrial applications.

We also investigated the pH and thermal stability of both free and immobilized enzymes. LXTE@BsCR-SpyTag exhibited remarkable pH stability, retaining over 80% relative activity after 24 h of incubation at pH 8.0 (Figure 5e). In contrast, the free BsCR enzyme activity dropped to only 50% under the same conditions (Figure S5a). Similarly, LXTE@BsGDH-SnoopTag displayed good stability across a broader pH range (pH 5.0–7.0), maintaining over 80% relative activity after 16 h of incubation (Figure 5f). The free BsGDH-SnoopTag enzyme only showed good stability at pH 7.0 (Figure S5b). These findings suggested that immobilization significantly enhances the pH stability of both enzymes. Thermal stability was also significantly improved by immobilization. After incubation at 25 °C for 8 h, LXTE@BsCR-SpyTag retained over 78% of its relative activity (Figure 5g), while LXTE@BsGDH-SnoopTag exhibited even greater stability, maintaining over 88% of its activity (Figure 5h). These results demonstrated a substantial increase in thermal stability compared to the free enzymes, as shown in the Supplementary Figures (Figure S5c,d). Furthermore, the immobilized enzymes displayed improved thermal stability even at higher temperatures.

### 3.5. Optimization of Parameters in Coupling Reaction

Although the enzyme activity analysis showed the optimal pH and temperature for each enzyme, the catalytic reaction requires the synergistic action of both enzymes. Therefore, exploring the optimal reaction conditions for the two-enzyme reaction system is crucial. We investigated free dual enzymes (BsCR-SpyTag and BsGDH-SnoopTag) and immobilized dual enzymes (LXTE@BsCR-SpyTag and LXTE@BsGDH-SnoopTag), respectively. Both free and immobilized dual-enzyme systems were prepared at a 1:1 molar ratio of the two enzymes, assuming equal importance for their activities in the overall reaction.

**Figure 6:**
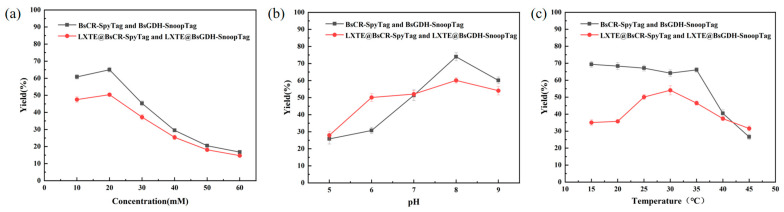
(**a**) Effect of substrate concentration on catalytic reaction; (**b**) Effect of pH on catalytic reaction; (**c**) Effect of reaction temperature on catalytic reaction.

The efficiency of the COBE asymmetric reduction reaction catalyzed by the free enzyme or the immobilized dual enzyme was compared by (S)-CHBE yield. Reaction conditions such as substrate concentration, pH, and temperature were optimized. Substrate concentration is essential for enzyme catalysis. At low concentration, the enzyme catalytic efficiency is limited by the availability of substrate. Conversely, high concentrations can lead to enzyme inhibition or even poisoning, hindering its catalytic ability. Therefore, optimizing substrate concentration is critical for maximizing enzyme performance. As shown in Figure 6a, both free and immobilized enzymes exhibited the highest productivity at a substrate concentration of 20 mM.

As depicted in Figure 6b, the yield of (S)-CHBE initially increased and then decreased with increasing buffer pH for both free and immobilized enzymes. For free and immobilized enzymes, the yield of (S)-CHBE peaked at a reaction pH of 8.0.

We performed COBE asymmetric reduction reactions at different temperatures to determine the optimal reaction temperature. As shown in Figure 6c, the yield of (S)-CHBE produced by the free enzyme remained stable between 15–35 °C but decreased significantly above 40 °C, possibly due to thermal denaturation of the enzyme. The immobilized enzyme achieved a maximum yield of 54.1% at 30 °C. Interestingly, the immobilized enzyme maintained a higher (S)-CHBE yield at 45 °C than the free enzyme. This suggested that immobilization may enhance enzyme stability by restricting conformational changes or providing a protective microenvironment, thereby mitigating thermal denaturation. Overall, the immobilized double-enzyme system reached a peak yield of 60.1% for (S)-CHBE under optimal conditions of 30 °C, pH 8.0, and 20 mM substrate.

### 3.6. Reusability of Immobilized Enzymes

**Figure 7:**
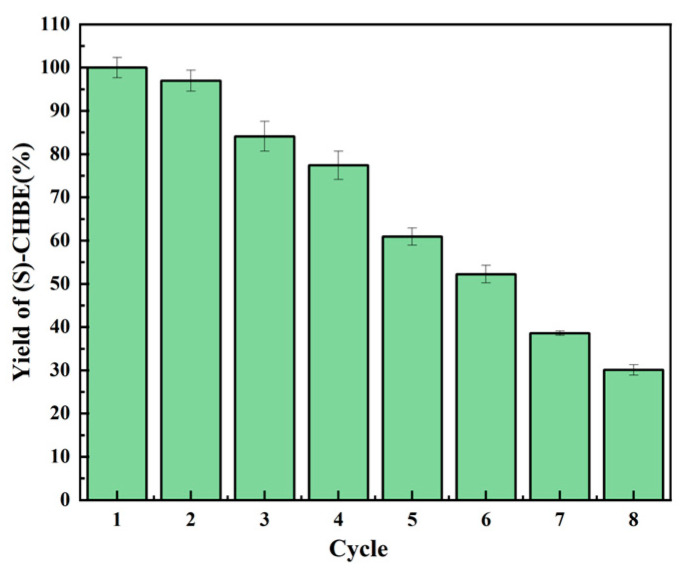
Reusability of LXTE@BsCR-SpyTag and LXTE@BsGDH-SnoopTag.

Compared with free enzymes, immobilized enzymes offer significant advantages in reusability, making them more attractive for practical applications. An immobilized enzyme’s stable catalytic performance allows it to maintain high activity over multiple reactions, significantly improving the operability and economic benefits of the biocatalysis process. We determined the reusability of the immobilized double-enzyme system consisting of LXTE@BsCR-SpyTag and LXTE@BsGDH-SnoopTag. As shown in Figure 7, the reusability of the immobilized enzyme was measured by the yield of (S)-CHBE in a continuous batch reaction. After the second cycle, the yield of (S)-CHBE remained as high as 96% of the initial yield. Even after six cycles, the yield of (S)-CHBE still maintained more than 50% of the initial yield, and after eight cycles of reuse, the yield of (S)-CHBE was 30.09%. Covalent immobilization technology is unique to enzyme engineering due to its excellent operational stability. Its key advantage is that it can effectively mitigate enzyme inactivation during biotransformation and significantly reduce enzyme leakage from the support. The high stability translates to extended enzyme lifespan and reduced operational costs, ultimately enhancing the economic feasibility of biocatalysis processes.

## 4. Discussion

This study demonstrated the effectiveness of Spy/SnoopTag and Spy/SnoopCatcher systems for one-step enzyme immobilization in the biocatalytic synthesis of (S)-CHBE. The formation of irreversible covalent bonds between Tags and Catcher ensures a stable connection between the enzyme and the carrier. This contrasts with reversible linkages, which can lead to enzyme leakage from the support [24,35]. Compared to other bioaffinity domains used for one-step protein purification and immobilization, such as chitin-binding domains [36] and cellulose-binding domains [37], the small sizes of SpyTag (only 16 residues) and SnoopTag (only 12 residues) minimize their impact on protein expression and folding.

Improving the thermal stability of enzymes is essential for their use in industry [38]. Our study revealed that the immobilized dual-enzyme system exhibited an increased yield of (S)-CHBE at higher reaction temperatures than the free enzyme system. This suggested that the immobilization method promotes enzyme activity under a wider range of conditions. Furthermore, research showed that SpyCatcher/SpyTag-mediated cyclization can significantly enhance enzyme thermal resistance, even to boiling temperatures [39]. In addition, the cyclized enzymes showed improved tolerance towards heavy metal ions, organic solvents, and denaturing *agents* [40]. Therefore, the co-immobilization of cyclized enzymes would be a promising step in the practical application of multiple enzymes.

Despite these advantages, some challenges warrant further investigation. The results demonstrated that the activity of the enzyme decreases after immobilization by the Spy/Snoop system. The reason could be that the unsealed epoxy groups on the carrier are interfering with the enzyme’s active site or reduced flexibility hindering substrate binding [41]. In our COBE asymmetric reduction process, substrate and product inhibition potentially contributed to lower substrate concentrations than those reported elsewhere [42]. To address this, an aqueous-organic two-phase system can be used to increase the substrate concentration and yield.

This flexible and efficient strategy also applies to other double-enzyme systems, showing broad application prospects. However, for successive cascades involving three or more components, more sophisticated strategies or approaches may be necessary to achieve effective enzyme immobilization. In addition, certain enzymes with complex high-level structures may be difficult to assemble using this method because the potential effects of fusion tags on their activity and aggregation still need to be discovered. Therefore, it is particularly essential to design and construct assembly components reasonably in multi-enzyme assembly [43]. This affinity-assisted covalent self-assembly on enzyme-epoxy support offers a simple and efficient strategy despite limitations for specific enzymes. It allows the epoxy carrier to capture and assemble the target enzyme directly from the cell lysate, simplifying the enzyme purification step. The strategy is expected to be applied to a wider range of enzyme immobilization scenarios through further optimization and improvement.

## 5. Conclusions

Ethyl (S)-4-chloro-3-hydroxybutyrate is an important chiral intermediate in the synthetic route of atorvastatin calcium, a best-selling drug in treating cardiovascular diseases [42]. It is of great practical significance to study the use of the SpyTag/SpyCatcher system and SnoopTag/SnoopCatcher system to realize the asymmetric reduction reaction and the directed coupling immobilization of coenzyme regeneration to realize the efficient synthesis of (S)-CHBE. In this study, two fusion proteins, BsCR-SpyTag and BsGDH-SnoopTag, were successfully constructed. The enzyme immobilization technique was skillfully integrated with the in vitro reaction of SpyTag/SpyCatcher and SnoopTag/SnoopCatcher to achieve one-step purification of the tagged fusion proteins. Two tagged enzymes were accurately immobilized on the epoxy resin. We constructed a double-enzyme directed fixation system for carbonyl reductase (BsCR) and glucose dehydrogenase (BsGDH). This system successfully achieved the asymmetric reduction reaction of COBE and facilitated coenzyme regeneration. Uniquely, we exploited the excellent specific binding ability between the Tag/Catcher protein pairs and could accurately capture and immobilize the target enzymes, significantly reducing purification costs. Immobilization by the Spy and Snoop systems not only simplifies the procedure but also enhances the optimal pH and temperature of the immobilized enzymes, allowing them to maintain high activity over a broader temperature range. In addition, this double-enzyme immobilization system can maintain high operational stability after multiple cycles of use, and the yield can still be maintained at about 50% of the initial yield after six cycles, which provides new possibilities for the development of biocatalysis. Through the “tag” modification of the fixed carrier and the target enzymes, the fixed carrier could quickly capture the target enzyme molecules from the crude enzyme solution, which realized the efficient and directional fixation of BsCR and BsGDH, and laid a foundation for the industrial production and application of (S)-CHBE.

## Figures and Tables

**Table 1:** Primers used in this study.

Primer Name	Primer Sequences (5′ to 3′)
yueD-F	CGCGCGCTCGAGCTACTACAAAAACTCTTTAATATCATAAATGCG
yueD-R	CGCGCGGGATCCATGGAACTTTATATCATCACCGGAG
bs-F	CGCGGATCCATGTATCCGGATTTAAAAGGAAA
bs-R	CGCCTCGAGTTAACCGCGGCCTGCCTGGA
YSTY44-R	GCATTTATGATATTAAAGAGTTTTTGGGAGGCTCCGGATCCGCT
YSTY56-F	GGATCTCAGTGGTGGTGGTGGTGGTGCTCGAGTTATTATTTGTAACGTTTATACGC
GSTY44-F	TGGTGGTGGTGGTGGTGCTCGAGTTACTTGTTGACCTTGATGAA
GSTY44-R	ATCCTTCATTCCAGGCAGGCCGCGGTGAATTCGCCAGAACCAGC

**Table 2:** Plasmids used and constructed in this study.

Plasmids	Purpose	Source
pET28a	Expression Vector	Our Laboratory
pET28a-*VLT*	Template	Our Laboratory
pET28a-*DLSN*	Template	Our Laboratory
pET28a-His-*PT-SpyCatcher*	SpyCatcher Expression Vector	Our Laboratory
pET28a-His-*PT-SnoopCatcher*	SnoopCatcher Expression Vector	Our Laboratory
pET28a-*yueD*	Carbonyl Reductase Expression Vector	This Study
pET28a-*bsgdh*	Glucose Dehydrogenase Expression Vector	This Study
pET28a-*ydlspt*	Fusion Expression Vectors Carbonyl Reductase and SpyTag (SpyTag in C Segment)	This Study
pET28a-*bsgdhsnpt*	Fusion Expression Vectors Glucose Dehydrogenase and SnoopTag (SnoopTag in C Segment)	This Study

**Table 3:** Enzyme activity of free enzymes and the immobilized enzymes.

Enzyme	Activity (U/mg)
BscR	1.986
LXTE@BsCR-SpyTag	0.669
BsGDH	9.037
LXTE@BsGDH-SnoopTag	2.836

## Data Availability

Data are contained within the article and Supplementary Materials.

## Supplementary Materials

The following supporting information can be downloaded at: https://www.mdpi.com/article/10.3390/biom14040504/s1, Figure S1. The plasmidprofiles. (a) pET28a-VLT. (b) pET28a-DLSN. (c) pET28a-SpyCatcher. (d) pET28a-SnoopCatcher. Figure S2. SDS-PAGE analysis of recombinant fusion protein expression in *E. coli*. (a) M: Protein Marker; lane 1: BsCR crude enzyme. (b) M: Protein Marker; lane 1: Purified BsCR enzyme solution. (c) M: Protein Marker; lane 1: BsGDH crude enzyme; lane 2: Purified BsGDH enzyme solution. Figure S3. SDS-PAGE results of immobilization of purified SpyCatcher and SnoopCatcher; M: Protein Marker; lane 1: Purified SpyCatcher; lane 2–4: The SpyCatcher supernatant occurred after fixation; lane 5: Purified SnoopCatcher; lane 6–8: SnoopCatcher after fixed supernatant. Figure S4. FT-IR spectra of the epoxy resin and the immobilized resin. Figure S5. Effects of pH and temperature on BsCR-SpyTag and BsGDH-SnoopTag stability. (a) PH Stability of BsCR-SpyTag: various buffers of PH 5.0–9.0 for 24 h at 4 °C. (b) The pH stability of BsGDH-SnoopTag: various buffers of pH 5.0–9.0 for 24 h at 4 °C. (c) Temperature stability of BsCR-SpyTag: 25 °C, 30 °C, 35 °C, 40 °C and 45 °C, pH 7.0, 24 h. (d) Temperature stability of BsGDH-SnoopTag: 25 °C, 30 °C, 35 °C, 40 °C and 45 °C, pH 7.0, 24 h. Table S1. Nucleic acid sequences of constructed fusion proteins. Table S2. Amino acids sequences of constructed fusion proteins. Table S3. Immobilization efficiency of the BsCR-SpyTag and BsGDH-SnoopTag.

## References

[1] Ye Q., Ouyang P., Ying H. (2011). A review—Biosynthesis of optically pure ethyl (S)-4-chloro-3-hydroxybutanoate ester: Recent advances and future perspectives. Appl. Microbiol. Biotechnol..

[2] Kaliaperumal T., Kumar S., Gummadi S.N., Chadha A. (2010). Asymmetric synthesis of (S)-ethyl-4-chloro-3-hydroxybutanoate using Candida parapsilosis ATCC 7330. J. Ind. Microbiol. Biotechnol..

[3] Wan N.W., Liu Z.Q., Xue F., Shen Z.Y., Zheng Y.G. (2015). A One-Step Biocatalytic Process for (S)-4-Chloro-3-hydroxybutyronitrile using Halohydrin Dehalogenase: A Chiral Building Block for Atorvastatin. ChemCatChem.

[4] Steinreiber J., Faber K., Griengl H. (2008). De-racemization of Enantiomers versus De-epimerization of Diastereomers—Classification of Dynamic Kinetic Asymmetric Transformations (DYKAT). Chem.–A Eur. J..

[5] Wu J., Pai C.C., Kwok W.H., Guo R.W., Au-Yeung T.T., Yeung C.H., Chan A.S. (2003). Studies on the rhodium- and ruthenium-catalyzed asymmetric hydrogenation of α-dehydroamino acids using a family of chiral dipyridylphosphine ligand (P-Phos). Tetrahedron Asymmetry.

[6] Zheng G., Xu J. (2011). New opportunities for biocatalysis: Driving the synthesis of chiral chemicals. Curr. Opin. Biotechnol..

[7] Naeem M., Li A., Younis M.A., Shen B., Ye L., Yu H. (2019). Asymmetric Bioreduction of 4-hydroxy-2-butanone by Carbonyl Reductases PFODH and CpSADH Delivers 1,3-butanediol Enantiomers with Excellent R- and S-enantioselectivity. Biotechnol. Bioprocess Eng..

[8] Tan Z., Ma H., Li Q., Pu L., Cao Y., Qu X., Zhu C., Ying H. (2016). Biosynthesis of optically pure chiral alcohols by a substrate coupled and biphasic system with a short-chain dehydrogenase from Streptomyces griseus. Enzym. Microb. Technol..

[9] Wang Q., Shen L., Ye T., Cao D., Chen R., Pei X., Xie T., Li Y., Gong W., Yin X. (2012). Overexpression and characterization of a novel (S)-specific extended short-chain dehydrogenase/reductase from Candida parapsilosis. Bioresour. Technol..

[10] Wang Q., Ye T., Ma Z., Chen R., Xie T., Yin X. (2014). Characterization and site-directed mutation of a novel aldo–keto reductase from Lodderomyces elongisporus NRRL YB-4239 with high production rate of ethyl (R)-4-chloro-3-hydroxybutanoate. J. Ind. Microbiol. Biotechnol..

[11] Zhao H., van der Donk W.A. (2003). Regeneration of cofactors for use in biocatalysis. Curr. Opin. Biotechnol..

[12] Yang H., Jia X., Han Y. (2022). Microbial redox coenzyme engineering and applications in biosynthesis. Trends Microbiol..

[13] Betancor L., Berne C., Luckarift H.R., Spain J.C. (2006). Coimmobilization of a redox enzyme and a cofactor regeneration system. Chem. Commun..

[14] Liu W., Wang P. (2007). Cofactor regeneration for sustainable enzymatic biosynthesis. Biotechnol. Adv..

[15] Rao J., Zhang R., Xu G., Li L., Xu Y. (2020). Efficient production of (S)-1-phenyl-1,2-ethanediol using xylan as co-substrate by a coupled multi-enzyme Escherichia coli system. Microb. Cell Factories.

[16] Jakoblinnert A., Mladenov R., Paul A., Sibilla F., Schwaneberg U., Ansorge-Schumacher M.B., de María P.D. (2011). Asymmetric reduction of ketones with recombinant *E. coli* whole cells in neat substrates. Chem. Commun..

[17] Gröger H., Chamouleau F., Orologas N., Rollmann C., Drauz K., Hummel W., Weckbecker A., May O. (2006). Enantioselective Reduction of Ketones with “Designer Cells” at High Substrate Concentrations: Highly Efficient Access to Functionalized Optically Active Alcohols. Angew. Chem. Int. Ed..

[18] Zhou L., Ouyang Y., Kong W., Ma T., Zhao H., Jiang Y., Gao J., Ma L. (2022). One pot purification and co-immobilization of His-tagged old yellow enzyme and glucose dehydrogenase for asymmetric hydrogenation. Enzym. Microb. Technol..

[19] Garcia-Bofill M., Sutton P.W., Guillen M., Alvaro G. (2021). Enzymatic synthesis of a statin precursor by immobilised alcohol dehydrogenase with NADPH oxidase as cofactor regeneration system. Appl. Catal. A: Gen..

[20] Peng F., Ou X.Y., Guo Z.W., Zeng Y.J., Zong M.H., Lou W.Y. (2020). Co-immobilization of multiple enzymes by self-assembly and chemical crosslinking for cofactor regeneration and robust biocatalysis. Int. J. Biol. Macromol..

[21] Schoffelen S., van Hest J.C. (2013). Chemical approaches for the construction of multi-enzyme reaction systems. Curr. Opin. Struct. Biol..

[22] Jiang Y., Zhang X., Yuan H., Huang D., Wang R., Liu H., Wang T. (2021). Research progress and the biotechnological applications of multienzyme complex. Appl. Microbiol. Biotechnol..

[23] Zakeri B., Fierer J.O., Celik E., Chittock E.C., Schwarz-Linek U., Moy V.T., Howarth M. (2012). Peptide tag forming a rapid covalent bond to a protein, through engineering a bacterial adhesin. Proc. Natl. Acad. Sci. USA.

[24] Reddington S.C., Howarth M. (2015). Secrets of a covalent interaction for biomaterials and biotechnology: SpyTag and SpyCatcher. Curr. Opin. Chem. Biol..

[25] Izoré T., Contreras-Martel C., El Mortaji L., Manzano C., Terrasse R., Vernet T., Di Guilmi A.M., Dessen A. (2010). Structural Basis of Host Cell Recognition by the Pilus Adhesin from Streptococcus pneumoniae. Structure.

[26] Buldun C.M., Jean J.X., Bedford M.R., Howarth M. (2018). SnoopLigase Catalyzes Peptide–Peptide Locking and Enables Solid-Phase Conjugate Isolation. J. Am. Chem. Soc..

[27] Qu J., Cao S., Wei Q., Zhang H., Wang R., Kang W., Ma T., Zhang L., Liu T., Wing-Ngor Au S. (2019). Synthetic Multienzyme Complexes, Catalytic Nanomachineries for Cascade Biosynthesis In Vivo. ACS Nano.

[28] Bao J., Liu N., Zhu L., Xu Q., Huang H., Jiang L. (2018). Programming a Biofilm-Mediated Multienzyme-Assembly-Cascade System for the Biocatalytic Production of Glucosamine from Chitin. J. Agric. Food Chem..

[29] Lin Y., Jin W., Cai L., Liu X., Qiu Y., Zhang G. (2021). Green preparation of covalently co-immobilized multienzymes on silica nanoparticles for clean production of reducing sugar from lignocellulosic biomass. J. Clean. Prod..

[30] Wei Q., He S., Qu J., Xia J. (2020). Synthetic Multienzyme Complexes Assembled on Virus-like Particles for Cascade Biosynthesis In Cellulo. Bioconjugate Chem..

[31] Li L., Fierer J.O., Rapoport T.A., Howarth M. (2014). Structural Analysis and Optimization of the Covalent Association between SpyCatcher and a Peptide Tag. J. Mol. Biol..

[32] Khairil Anuar I.N., Banerjee A., Keeble A.H., Carella A., Nikov G.I., Howarth M. (2019). Spy&Go purification of SpyTag-proteins using pseudo-SpyCatcher to access an oligomerization toolbox. Nat. Commun..

[33] Zou S.P., Wang Z.C., Qin C., Zheng Y.G. (2016). Covalent immobilization of Agrobacterium radiobacter epoxide hydrolase on ethylenediamine functionalised epoxy supports for biocatalytical synthesis of (R)-epichlorohydrin. Biotechnol. Lett..

[34] Wang J., Lu Y., Cheng P., Zhang C., Tang L., Du L., Li J., Ou Z. (2023). Construction of Bi-Enzyme Self-Assembly Clusters Based on SpyCatcher/SpyTag for the Efficient Biosynthesis of (R)-Ethyl 2-hydroxy-4-phenylbutyrate. Biomolecules.

[35] Ansari S.A., Husain Q. (2012). Potential applications of enzymes immobilized on/in nano materials: A review. Biotechnol. Adv..

[36] Chern J.-T., Chao Y.-P. (2005). Chitin-binding domain based immobilization of d-hydantoinase. J. Biotechnol..

[37] Linder M., Nevanen T., Söderholm L., Bengs O., Teeri T.T. (1998). Improved immobilization of fusion proteins via cellulose-binding domains. Biotechnol. Bioeng..

[38] Gajšek M., Jančič U., Vasić K., Knez Ž., Leitgeb M. (2019). Enhanced activity of immobilized transglutaminase for cleaner production technologies. J. Clean. Prod..

[39] Schoene C., Fierer J.O., Bennett S.P., Howarth M. (2014). SpyTag/SpyCatcher Cyclization Confers Resilience to Boiling on a Mesophilic Enzyme. Angew. Chem. Int. Ed..

[40] Wang Y., Chang Y., Jia R., Sun H., Tian J., Luo H., Yu H., Shen Z. (2020). SpyTag/SpyCatcher cyclization and covalent immobilization in enhancing cephalosporin C acylase stability. Process Biochem..

[41] Mateo C., Grazu V., Palomo J.M., Lopez-Gallego F., Fernandez-Lafuente R., Guisan J.M. (2007). Immobilization of enzymes on heterofunctional epoxy supports. Nat. Protoc..

[42] You Z.Y., Liu Z.Q., Zheng Y.G. (2014). Characterization of a newly synthesized carbonyl reductase and construction of a biocatalytic process for the synthesis of ethyl (S)-4-chloro-3-hydroxybutanoate with high space-time yield. Appl. Microbiol. Biotechnol..

[43] Tan Z., Cheng H., Chen G., Ju F., Fernández-Lucas J., Zdarta J., Jesionowski T., Bilal M. (2023). Designing multifunctional biocatalytic cascade system by multi-enzyme co-immobilization on biopolymers and nanostructured materials. Int. J. Biol. Macromol..

