# Supplementary material for: Catalytic Synthesis of (S)-CHBE by Directional Coupling and Immobilization of Carbonyl Reductase and Glucose Dehydrogenase
Source: Biomolecules. 2024 Apr 21;14(4):504. doi: 10.3390/biom14040504 (PMC11048691; doi:10.3390/biom14040504)
Supplement: Supplementary file 1 [file biomolecules-14-00504-s001.zip › biomolecules-2952920-supplementary.pdf]

Supplementary Materials

Catalytic Synthesis of (S)-CHBE by Directional Coupling and Immobilization of Carbonyl Reductase and Glucose Dehydrogenase

Yadong Wang [1,2,†], Ruiqi Sun [1,2,†], Peng Chen [1,2] and Fenghuan Wang [1,2,*]

[1] Key Laboratory of Geriatric Nutrition and Health (Beijing Technology and Business University), Ministry of Education, Beijing 100048, China; wangyadong@btbu.edu.cn (Y.W.); srqsp2017@163.com (R.S.); cp15212120947@163.com (P.C.)
[2] School of Light Industry Science and Engineering, Beijing Technology and Business University (BTBU), Beijing 100048, China
* Correspondence: wangfenghuan@th.btbu.edu.cn; Tel.: +86-10-68985252
† These authors contributed equally to this work.

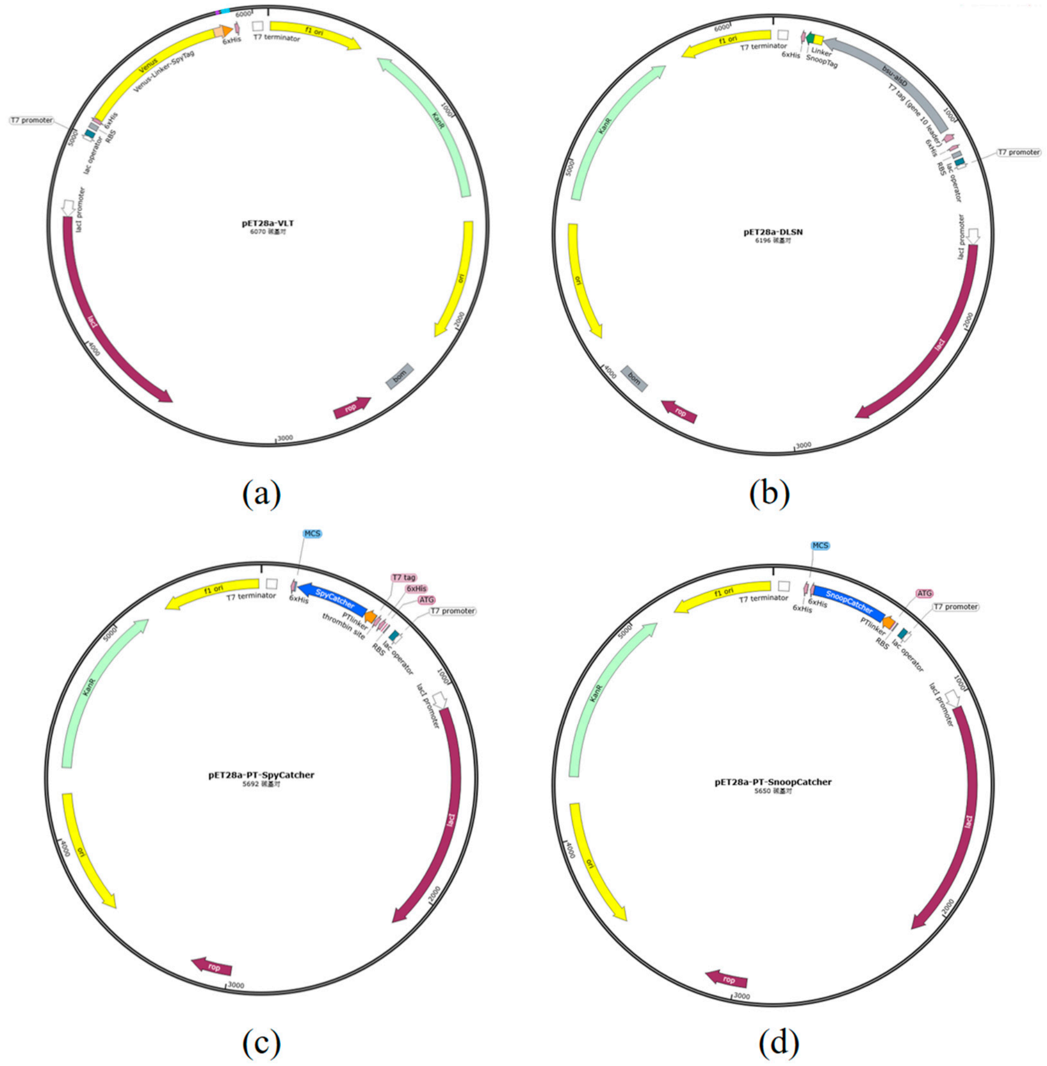

Figure S1. The plasmid profiles. (**a**) pET28a-VLT. (**b**) pET28a-DLSN. (**c**) pET28a-SpyCatcher. (**d**) pET28a-SnoopCatcher.

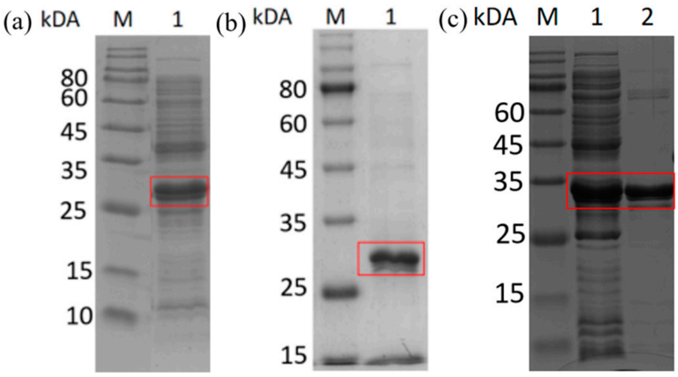

Figure S2. SDS-PAGE analysis of recombinant fusion protein expression in E. coli. (**a**) M: Protein Marker; lane 1: BsCR crude enzyme. (**b**) M: Protein Marker; lane 1: Purified BsCR enzyme solution. (**c**) M: Protein Marker; lane 1: BsGDH crude enzyme; lane 2: Purified BsGDH enzyme solution.

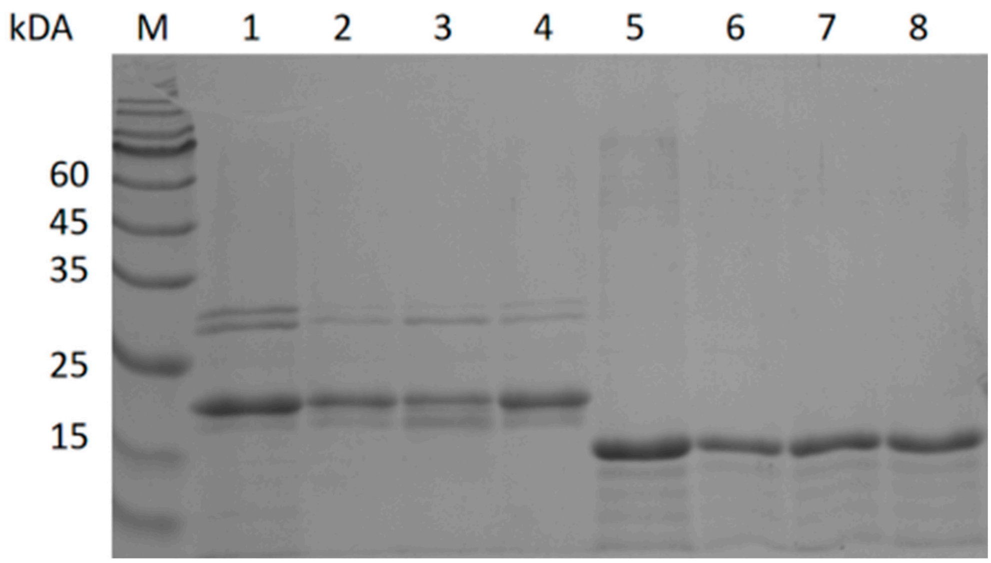

Figure S3. SDS-PAGE results of immobilization of purified SpyCatcher and SnoopCatcher ; M: Protein Marker; lane 1: Purified SpyCatcher; lane 2-4: The SpyCatcher supernatant occurred after fixation; lane 5: Purified SnoopCatcher; lane 6-8: SnoopCatcher After fixed supernatant.

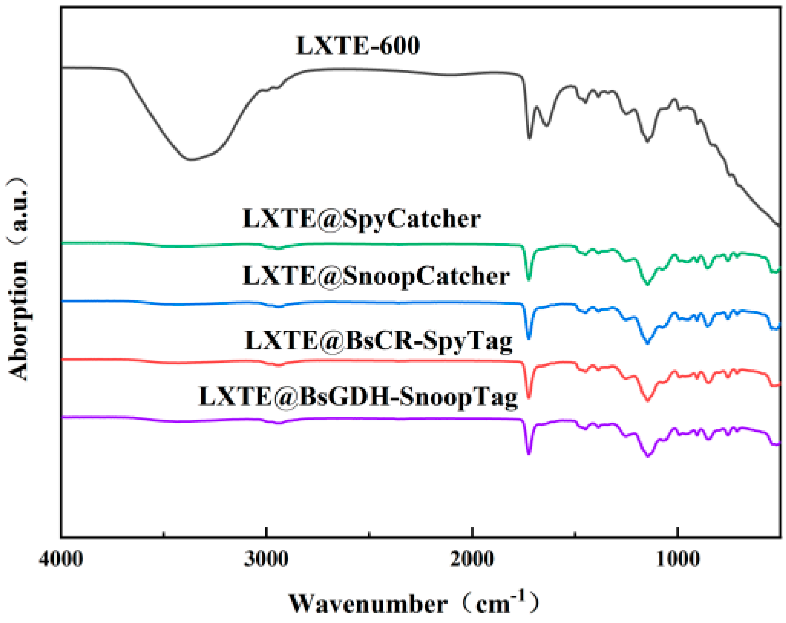

Figure S4. FT-IR spectra of the epoxy resin and the immobilized resin.

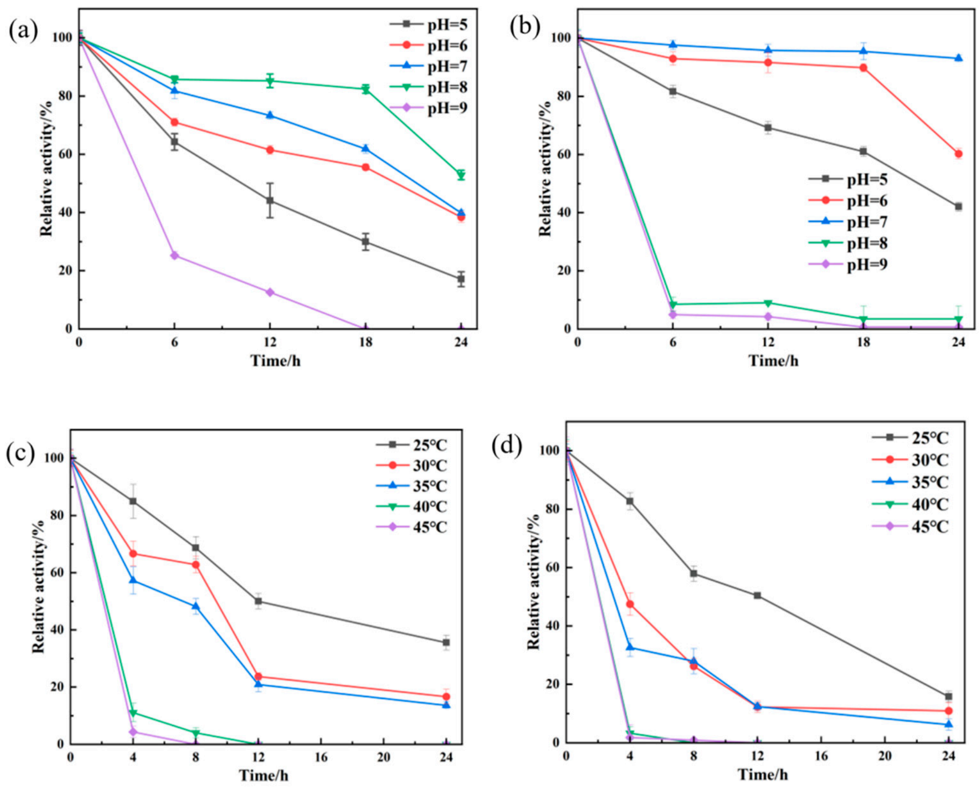

Figure S5. Effects of pH and temperature on BsCR-SpyTag and BsGDH-SnoopTag stability. (a) PH Stability of BsCR-SpyTag: various buffers of PH 5.0-9.0 for 24 hours at 4 ℃. (b) The pH stability of BsGDH-SnoopTag: various buffers of pH 5.0-9.0 for 24 hours at 4 ℃. (c) Temperature stability of BsCR-SpyTag: 25 ℃, 30 ℃, 35 ℃ ,40 ℃and 45 ℃, pH 7.0 , 24 hours. (d) Temperature stability of BsGDH-SnoopTag: 25 ℃, 30 ℃, 35 ℃ ,40 ℃and 45 ℃, pH 7.0 , 24 hours.

Table S1. Nucleic acid sequences of constructed fusion proteins:

Gene name	Nucleic acid sequences
Linker-SpyTag:	GGAGGCTCCGGATCCGCTGGCTCCGCTGCTGGTTCTGGCGAA TTCCGTGGCGTTCCGCACATCGTTATGGTTGATGCGTATAAAC GTTACAAA
Linker-SnoopTag:	GAATTCGCCAGAACCAGCAGCGGAGCCAGCGGATCCGGAGC CTCCAAGTTGGGTGACATCGAGTTCATCAAGGTCAACAAG
SpyCatcher:	GTTACCACCCTGAGCGGCCTGAGCGGTGAACAGGGCCCGAG CGGCGATATGACCACCGAAGAAGATAGCGCGACCCACATCA AATTCAGCAAACGTGATGAAGATGGCCGTGAACTGGCGGGC GCGACCATGGAACTGCGTGATAGCAGCGGTAAAACCATCAG CACCTGGATTAGTGACGGCCACGTGAAAGACTTTTATCTGTAT CCGGGTAAATATACCTTCGTTGAAACCGCTGCGCCGGATGGTT ACGAAGTTGCGACCCCGATCGAATTCACCGTTAACGAAGATG GTCAGGTTACCGTTGATGGTGAAGCGACCGAAGGTGATGCTC ACACC
SnoopCatcher:	AAACCGCTGCGTGGCGCGGTTTTCAGCCTGCAAAAACAGCA CCCGGATTACCCGGACATCTACGGCGCGATCGATCAGAACGG CACCTACCAGAACGTTCGTACCGGCGAAGATGGCAAACTGA

CCTTCAAAAACCTGAGCGATGGCAAATACCGTCTGTTCGAAA
ACAGCGAACCGGCGGGCTACAAACCGGTTCAGAACAAACCG
ATCGTTGCGTTCCAGATCGTTAACGGCGAAGTTCGTGATGTTA
CCAGCATCGTTCCGCAGGACATCCCGGCGACCTACGAATTTA
CCAACGGCAAACACTACATCACCAACGAACCGATCCCGCCG
AAA

yueD: ATGGAACTTTATATCATCACCGGAGCGTCAAAAGGGCTGGGT
CAAGCCATTGCATTACAGGCTTTAGAAAAGGGGCATGAAGTC
CATGCCTTATCCAGAACGAAAACAGATGTCTCTCACAAAAAA
CTAACGCAGCATCAAATAGACCTCATCAATCTCGAAGAAGCT
GAACAGCAATTTGAAACATTGCTCTCATCCATCGATTCAGATC
GTTATTCTGGTATTACCCTTATTAATAACGCCGGAATGGTAACG
CCGATCAAACGTGCCGGCGAAGCGTCTCTTGACGAGCTTCAG
CGCCATTATCAGCTGAACCTGACTGCGCCCGTGCTTTTGAGTC
AGCTGTTTACAAAACGGTTTGCTTCATACAGCGGCAAAAAGA
CGGTTGTCAACATTACTTCAGGAGCCGCCAAAAATCCATATA
AGGGATGGAGCGCGTATTGCAGTTCAAAAGCCGGGCTCGAC
ATGTTTACGAGGACATTCGGATTTGAACAGGAGGATGAAGAG
CTGCCGGTGAACATGATTTCGTTCTCACCTGGAGTGATGGAC
ACTGAGATGCAGGCCGTCATCCGTTCTTCATCGAAAAAGGAT
TTCCACCACATTGAACGATTCCGGAAATTAAATGAAACAGGA
AGCCTTCGCAGTCCGGACTTTATTGCCGGCACGCTGCTTTCTT
TACTAGAAAAAGGGACGGAAAACGGCCGCATTTATGATATTA
AAGAGTTTTTG

bsgdh: ATGTATCCGGATTTAAAAGGAAAAGTCGTCGCTATTACAGGA
GCTGCTTCAGGGCTCGGAAAGGCGATGGCCATTCGCTTCGGC
AAGGAGCAGGCAAAAGTGGTTATCAACTATTATAGTAATAAA
CAAGATCCGAACGAGGTAAAAGAAGAGGTCATCAAGGCGGG
CGGTGAAGCTGTTGTCGTCCAAGGAGATGTCACGAAAGAGG
AAGATGTAAAAAATATCGTGCAAACGGCAATTAAGGAGTTCG
GCACACTCGATATTATGATTAATAATGCCGGTCTTGAAAATCC
TGTGCCATCTCACGAAATGCCGCTCAAGGATTGGGATAAAGT
CATCGGCACGAACTTAACGGGTGCCTTTTTAGGAAGCCGTGA
AGCGATTAAATATTTCGTAGAAAACGATATCAAGGGAAATGT
CATTAACATGTCCAGTGTGCACGAAGTGATTCCTTGGCCGTTA
TTTGTCCACTATGCGGCAAGTAAAGGCGGGATAAAGCTGATG
ACAGAAACATTAGCGTTGGAATACGCGCCGAAGGGCATTCGC
GTCAATAATATTGGGCCAGGTGCGATCAACACGCCAATCAAT
GCTGAAAAATTCGCTGACCCTAAACAGAAAGCTGATGTAGA
AAGCATGATTCCAATGGGATATATCGGCGAACCGGAGGAGAT
CGCCGCAGTAGCAGCCTGGCTTGCTTCGAAGGAAGCCAGCTA
CGTCACAGGCATCACGTTATTCGCGGACGGCGGTATGACACA
ATATCCTTCATTCCAGGCAGGCCGCGGT

yueD-Linker-SpyTag: ATGGAACTTTATATCATCACCGGAGCGTCAAAAGGGCTGGGT

	CAAGCCATTGCATTACAGGCTTTAGAAAAGGGGCATGAAGTC<br>CATGCCTTATCCAGAACGAAAACAGATGTCTCTCACAAAAAA<br>CTAACGCAGCATCAAATAGACCTCATCAATCTCGAAGAAGCT<br>GAACAGCAATTTGAAACATTGCTCTCATCCATCGATTCAGATC<br>GTTATTCTGGTATTACCCTTATTAATAACGCCGGAATGGTAACG<br>CCGATCAAACGTGCCGGCGAAGCGTCTCTTGACGAGCTTCAG<br>CGCCATTATCAGCTGAACCTGACTGCGCCCGTGCTTTTGAGTC<br>AGCTGTTTACAAAACGGTTTGCTTCATACAGCGGCAAAAAGA<br>CGGTTGTCAACATTACTTCAGGAGCCGCCAAAAATCCATATA<br>AGGGATGGAGCGCGTATTGCAGTTCAAAAGCCGGGCTCGAC<br>ATGTTTACGAGGACATTCGGATTTGAACAGGAGGATGAAGAG<br>CTGCCGGTGAACATGATTTCGTTCTCACCTGGAGTGATGGAC<br>ACTGAGATGCAGGCCGTCATCCGTTCTTCATCGAAAAAGGAT<br>TTCCACCACATTGAACGATTCCGGAAATTAAATGAAACAGGA<br>AGCCTTCGCAGTCCGGACTTTATTGCCGGCACGCTGCTTTCTT<br>TACTAGAAAAAGGGACGGAAAACGGCCGCATTTATGATATTA<br>AAGAGTTTTTGGGAGGCTCCGGATCCGCTGGCTCCGCTGCTG<br>GTTCTGGCGAATTCCGTGGCGTTCCGCACATCGTTATGGTTGA<br>TGCGTATAAACGTTACAAA
bsgdh-Linker-SnoopTag:	ATGTATCCGGATTTAAAAGGAAAAGTCGTCGCTATTACAGGA<br>GCTGCTTCAGGGCTCGGAAAGGCGATGGCCATTCGCTTCGGC<br>AAGGAGCAGGCAAAAGTGGTTATCAACTATTATAGTAATAAA<br>CAAGATCCGAACGAGGTAAAAGAAGAGGTCATCAAGGCGGG<br>CGGTGAAGCTGTTGTCGTCCAAGGAGATGTCACGAAAGAGG<br>AAGATGTAAAAAATATCGTGCAAACGGCAATTAAGGAGTTCG<br>GCACACTCGATATTATGATTAATAATGCCGGTCTTGAAAATCC<br>TGTGCCATCTCACGAAATGCCGCTCAAGGATTGGGATAAAGT<br>CATCGGCACGAACTTAACGGGTGCCTTTTTAGGAAGCCGTGA<br>AGCGATTAAATATTTCGTAGAAAACGATATCAAGGGAAATGT<br>CATTAACATGTCCAGTGTGCACGAAGTGATTCCTTGGCCGTTA<br>TTTGTCCACTATGCGGCAAGTAAAGGCGGGATAAAGCTGATG<br>ACAGAAACATTAGCGTTGGAATACGCGCCGAAGGGCATTCGC<br>GTCAATAATATTGGGCCAGGTGCGATCAACACGCCAATCAAT<br>GCTGAAAAATTCGCTGACCCTAAACAGAAAGCTGATGTAGA<br>AAGCATGATTCCAATGGGATATATCGGCGAACCGGAGGAGAT<br>CGCCGCAGTAGCAGCCTGGCTTGCTTCGAAGGAAGCCAGCTA<br>CGTCACAGGCATCACGTTATTCGCGGACGGCGGTATGACACA<br>ATATCCTTCATTCCAGGCAGGCCGCGGTGAATTCGCCAGAAC<br>CAGCAGCGGAGCCAGCGGATCCGGAGCCTCCAAGTTGGGTG<br>ACATCGAGTTCATcAAGGTCAACAAG

Table S2. Amino acids sequences of constructed fusion proteins:

Fusion protein name	Amino acids sequences

Linker-SpyTag:	GGSGSAGSAAGSGEFRGVPHIVMVDAYKRYK
Linker-SnoopTag:	EFARTSSGASGSGASKLGDIEFIKVNK
SpyCatcher:	MGSSHHHHHHSSGLVPRGSHMPTPPTTPTPPTTPTPTPVTTLSGLS<br>GEQGPSGDMTTEEDSATHIKFSKRDEDGRELAGATMELRDSSGKTI<br>STWISDGHVKDFYLYPGKYTFVETAAPDGYEVATPIEFTVNEDGQV<br>TVDGEATEGDAHT
SnoopCatcher:	PTPPTTPTPPTTPTPTPKPLRGAVFSLQKQHPDYPDIYGAIDQNGTY<br>QNVRTGEDGKLTFKNLSDGKYRLFENSEPAGYKPVQNKPIVAFQI<br>VNGEVRDVTSIVPQDIPATYEFTNGKHYITNEPIPPKHHHHHH
BsCR:	MGSSHHHHHHSSGLVPRGSHMASMTGGQQMGRGSMELYIITGAS<br>KGLGQAIALQALEKGHEVHALSRTKTDVSHKKLTQHQIDLINLEE<br>AEQQFETLLSSIDSDRYSGITLINNAGMVTPIKRAGEASLDELQRHY<br>QLNLTAPVLLSQLFTKRFASYSGKKTVVNITSGAAKNPYKGWSAY<br>CSSKAGLDMFTRTFGFEQEDEELPVNMISFSPGVMDTEMQAVIRSS<br>SKKDFHHIERFRKLNETGSLRSPDFIAGTLLSLLEKGTENGRIYDIKE<br>FL
BsGDH:	MGSSHHHHHHSSGLVPRGSHMASMTGGQQMGRGSMYPDLKGK<br>VVAITGAASGLGKAMAIRFGKEQAKVVINYYSNKQDPNEVKEEVI<br>KAGGEAVVVQGDVTKEEDVKNIVQTAIKEFGTLDIMINNAGLENP<br>VPSHEMPLKDWDKVIGTNLTGAFLGSREAIKYFVENDIKGNVINM<br>SSVHEVIPWPLFVHYAASKGGIKLMTETLALEYAPKGIRVNNIGPG<br>AINTPINAEKFADPKQKADVESMIPMGYIGEPEEIAAVAAWLASKE<br>ASYVTGITLFADGGMTQYPSFQAGRG
BsCR-SpyTag:	MGSSHHHHHHSSGLVPRGSHMASMTGGQQMGRGSMELYIITGAS<br>KGLGQAIALQALEKGHEVHALSRTKTDVSHKKLTQHQIDLINLEE<br>AEQQFETLLSSIDSDRYSGITLINNAGMVTPIKRAGEASLDELQRHY<br>QLNLTAPVLLSQLFTKRFASYSGKKTVVNITSGAAKNPYKGWSAY<br>CSSKAGLDMFTRTFGFEQEDEELPVNMISFSPGVMDTEMQAVIRSS<br>SKKDFHHIERFRKLNETGSLRSPDFIAGTLLSLLEKGTENGRIYDIKE<br>FLGGSGSAGSAAGSGEFRGVPHIVMVDAYKRYK
BsGDH-SnoopTag:	MGSSHHHHHHSSGLVPRGSHMASMTGGQQMGRGSMYPDLKGK<br>VVAITGAASGLGKAMAIRFGKEQAKVVINYYSNKQDPNEVKEEVI<br>KAGGEAVVVQGDVTKEEDVKNIVQTAIKEFGTLDIMINNAGLENP<br>VPSHEMPLKDWDKVIGTNLTGAFLGSREAIKYFVENDIKGNVINM<br>SSVHEVIPWPLFVHYAASKGGIKLMTETLALEYAPKGIRVNNIGPG<br>AINTPINAEKFADPKQKADVESMIPMGYIGEPEEIAAVAAWLASKE<br>ASYVTGITLFADGGMTQYPSFQAGRGEFARTSSGASGSGASKLGDI<br>EFIKVNK

Table S3. Immobilization efficiency of the BsCR-SpyTag and BsGDH-SnoopTag

Immobilization efficiency of the BsCR-SpyTag	Immobilization efficiency of the BsGDH-SnoopTag
69.24%	69.96%
